# Supplementary material for: Office of Admissions: Engagement and Leadership Opportunities for Trainees
Source: MedEdPORTAL. 2020 Nov 24;16:11018. doi: 10.15766/mep_2374-8265.11018 (PMC7703483; doi:10.15766/mep_2374-8265.11018)
Supplement: Supplementary file 1 — PowerPoint Presentation.pptxFacilitator Guide.docxPrereading Assignment.docxSkill-Set Group Mixer.docxAdmission Cases.docxPre- and Postworkshop Survey.docx [file mep_2374-8265.11018-s001.zip › A. PowerPoint Presentation.pptx]

## Slide 1
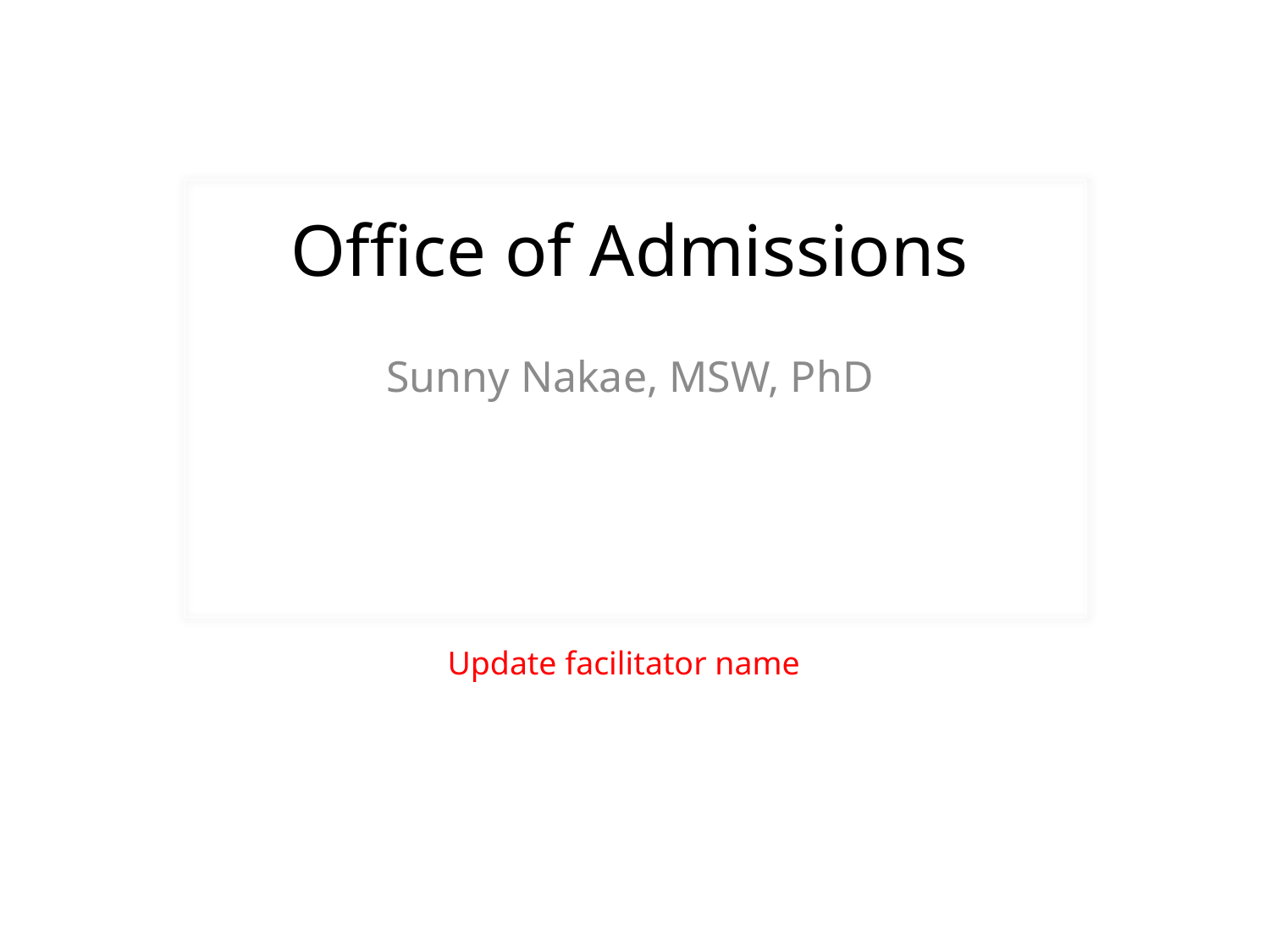

Office of Admissions
Sunny Nakae, MSW, PhD
Update facilitator name

## Slide 2
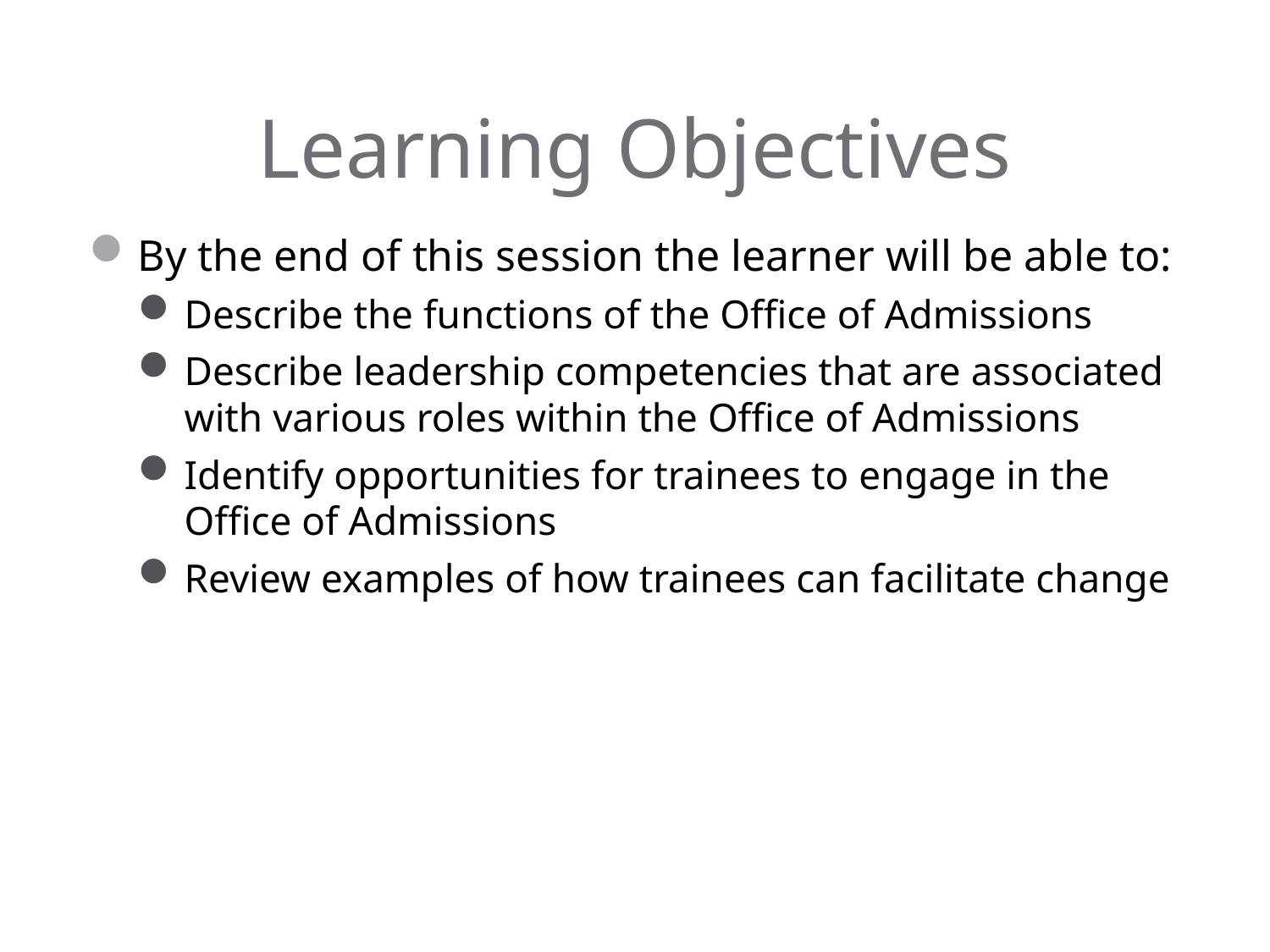

# Learning Objectives
By the end of this session the learner will be able to:
Describe the functions of the Office of Admissions
Describe leadership competencies that are associated with various roles within the Office of Admissions
Identify opportunities for trainees to engage in the Office of Admissions
Review examples of how trainees can facilitate change

## Slide 3
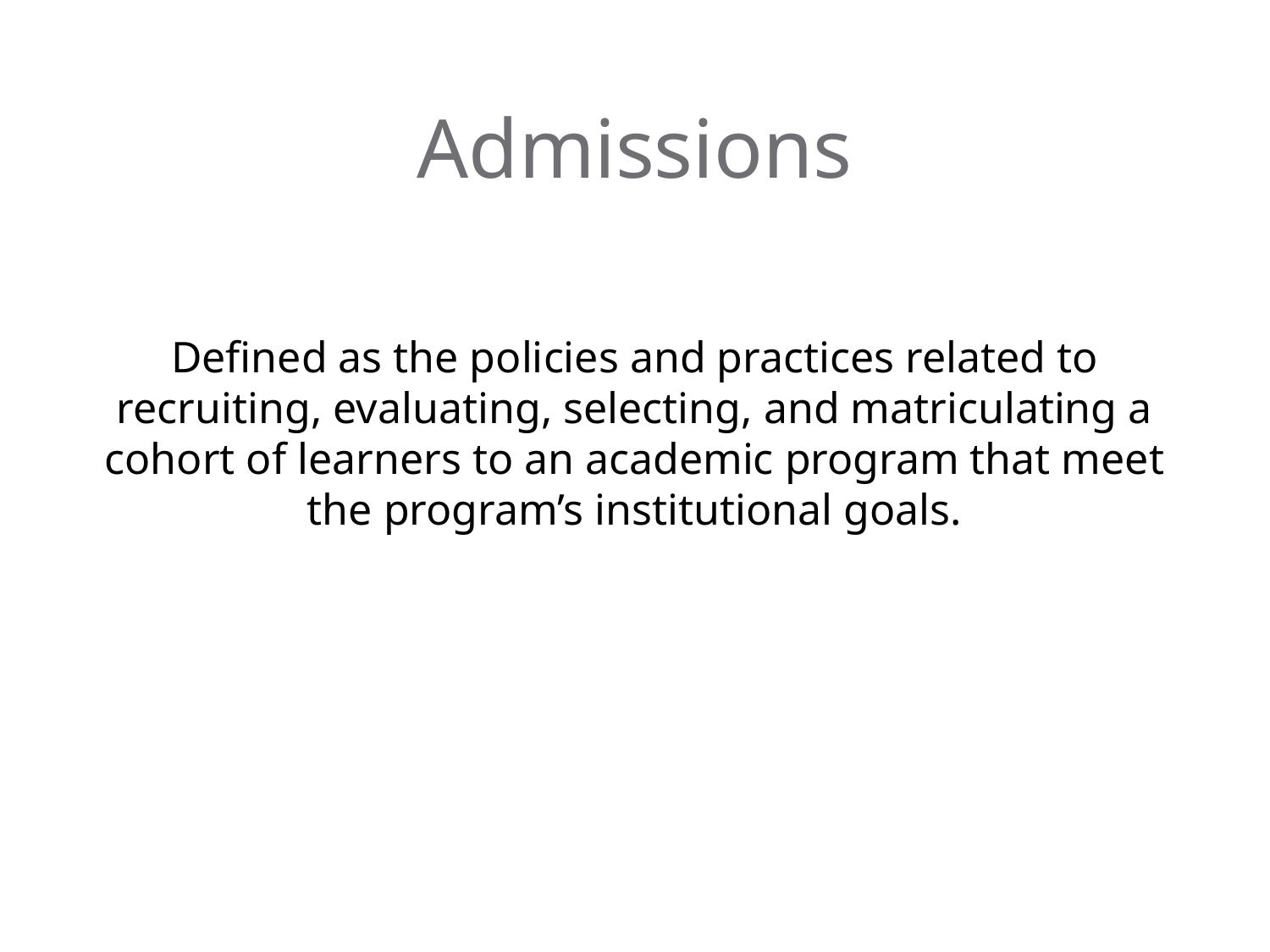

# Admissions
Defined as the policies and practices related to recruiting, evaluating, selecting, and matriculating a cohort of learners to an academic program that meet the program’s institutional goals.

## Slide 4
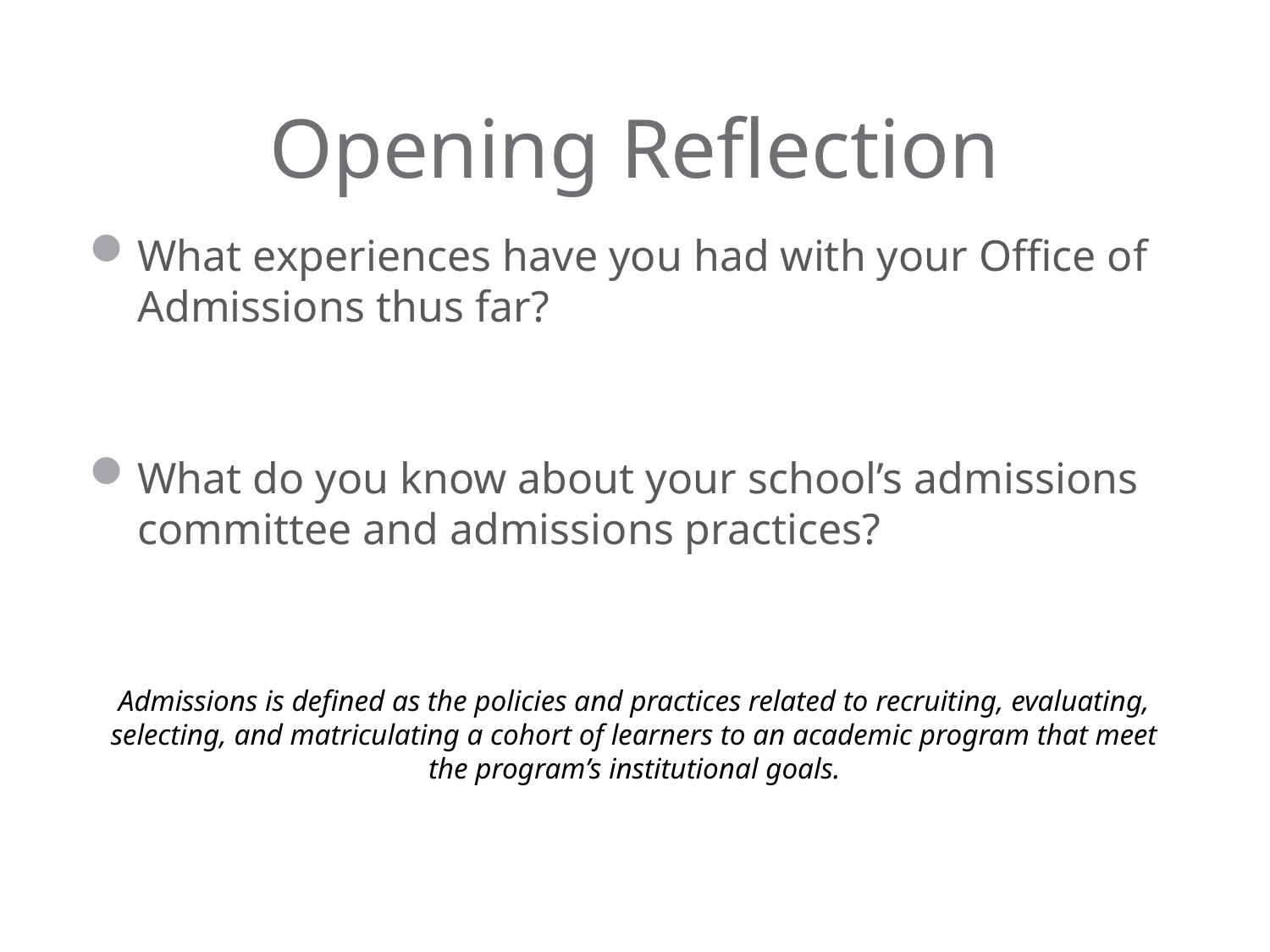

# Opening Reflection
What experiences have you had with your Office of Admissions thus far?
What do you know about your school’s admissions committee and admissions practices?
Admissions is defined as the policies and practices related to recruiting, evaluating, selecting, and matriculating a cohort of learners to an academic program that meet the program’s institutional goals.

## Slide 5
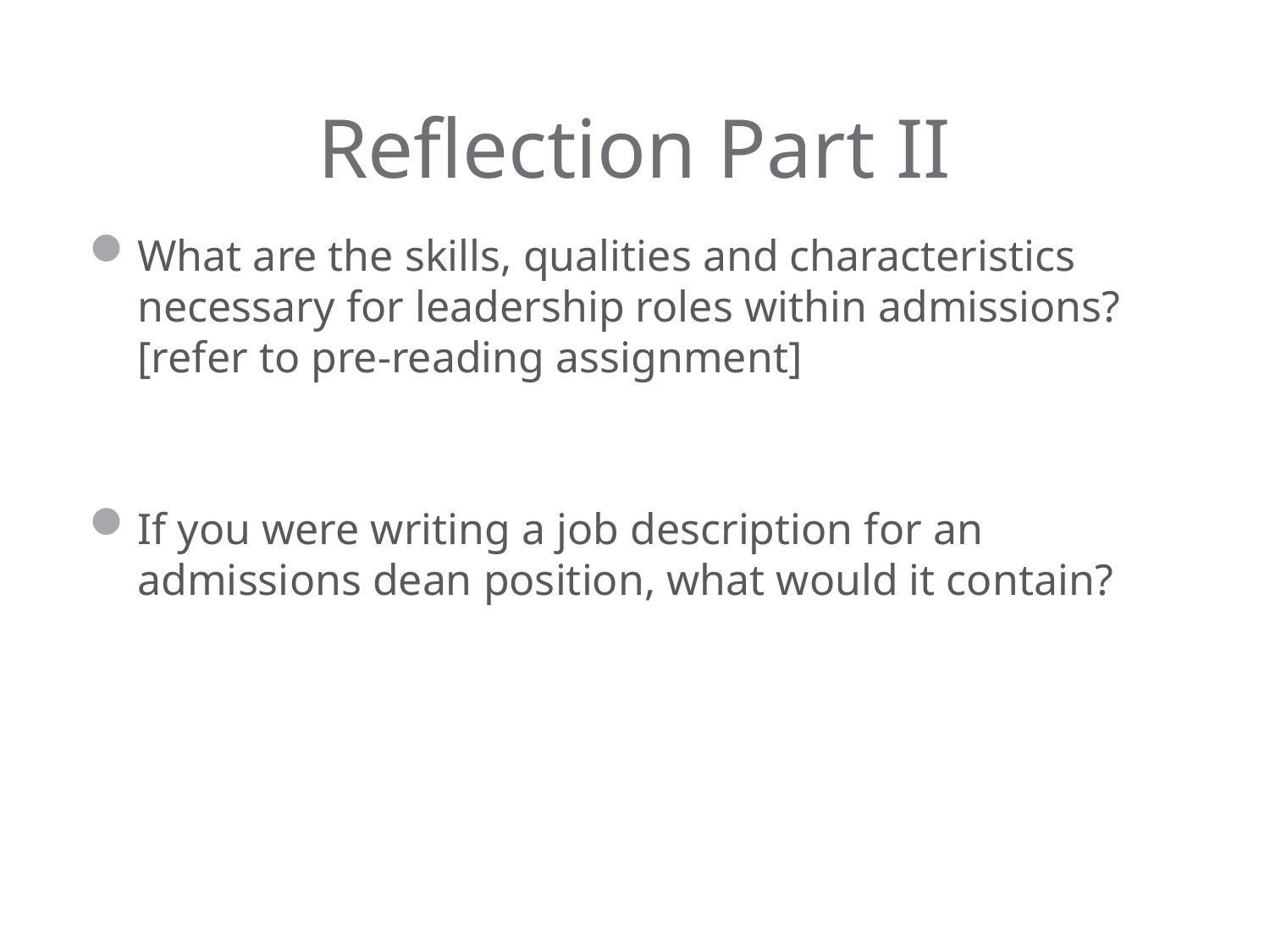

# Reflection Part II
What are the skills, qualities and characteristics necessary for leadership roles within admissions? [refer to pre-reading assignment]
If you were writing a job description for an admissions dean position, what would it contain?

## Slide 6
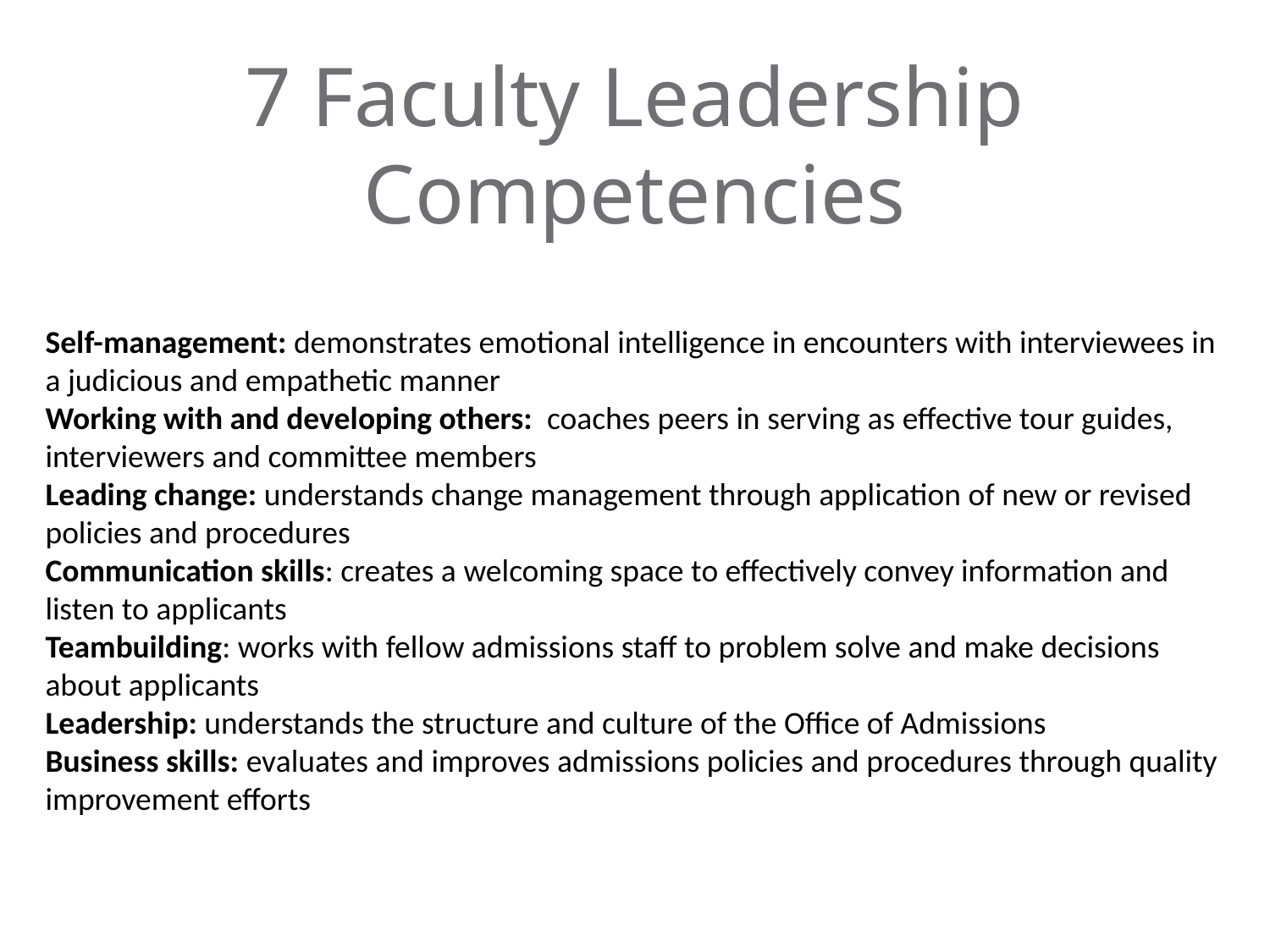

# 7 Faculty Leadership Competencies
Self-management: demonstrates emotional intelligence in encounters with interviewees in a judicious and empathetic manner
Working with and developing others: coaches peers in serving as effective tour guides, interviewers and committee members
Leading change: understands change management through application of new or revised policies and procedures
Communication skills: creates a welcoming space to effectively convey information and listen to applicants
Teambuilding: works with fellow admissions staff to problem solve and make decisions about applicants
Leadership: understands the structure and culture of the Office of Admissions
Business skills: evaluates and improves admissions policies and procedures through quality improvement efforts

## Slide 7
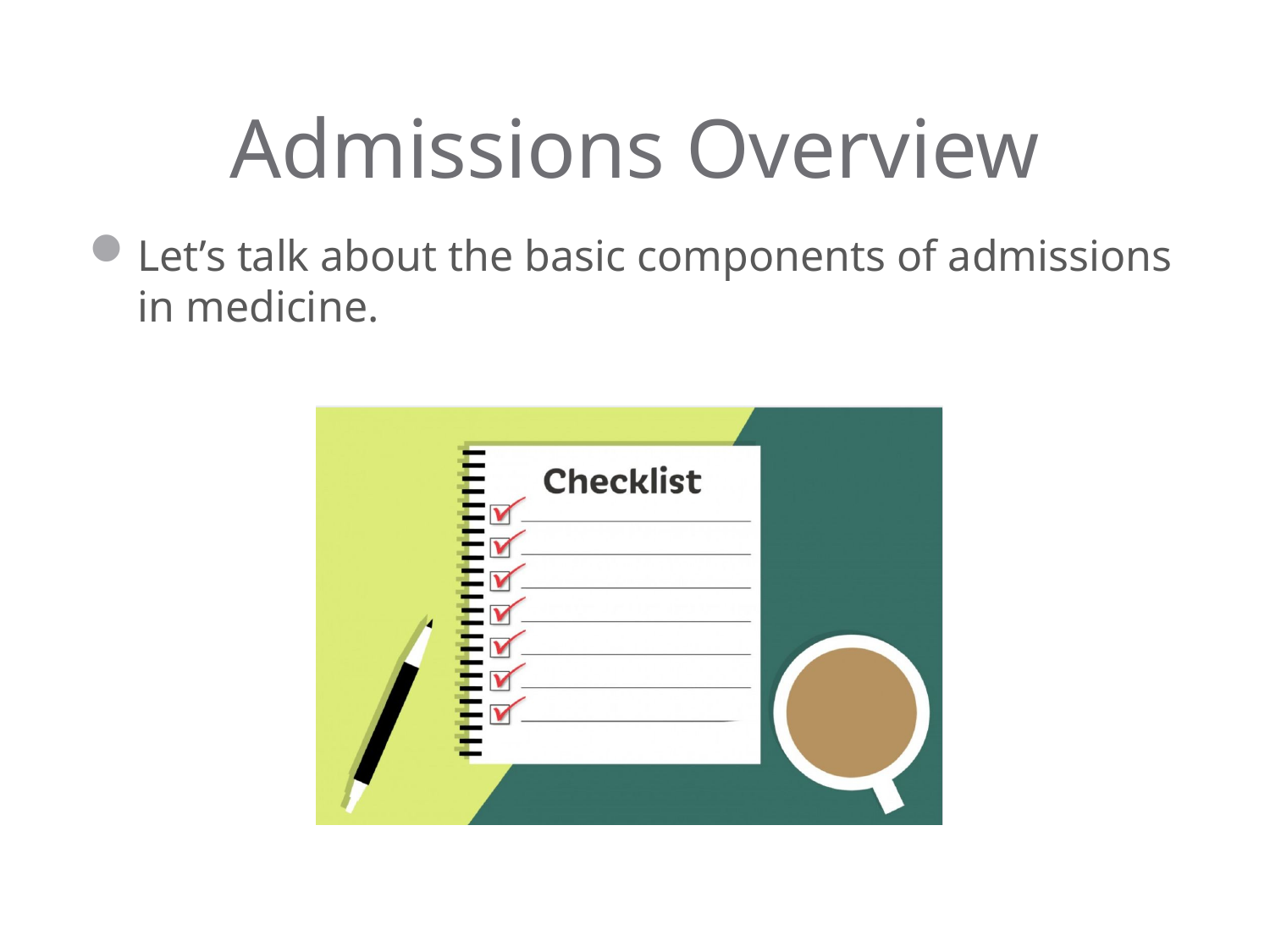

# Admissions Overview
Let’s talk about the basic components of admissions in medicine.

## Slide 8
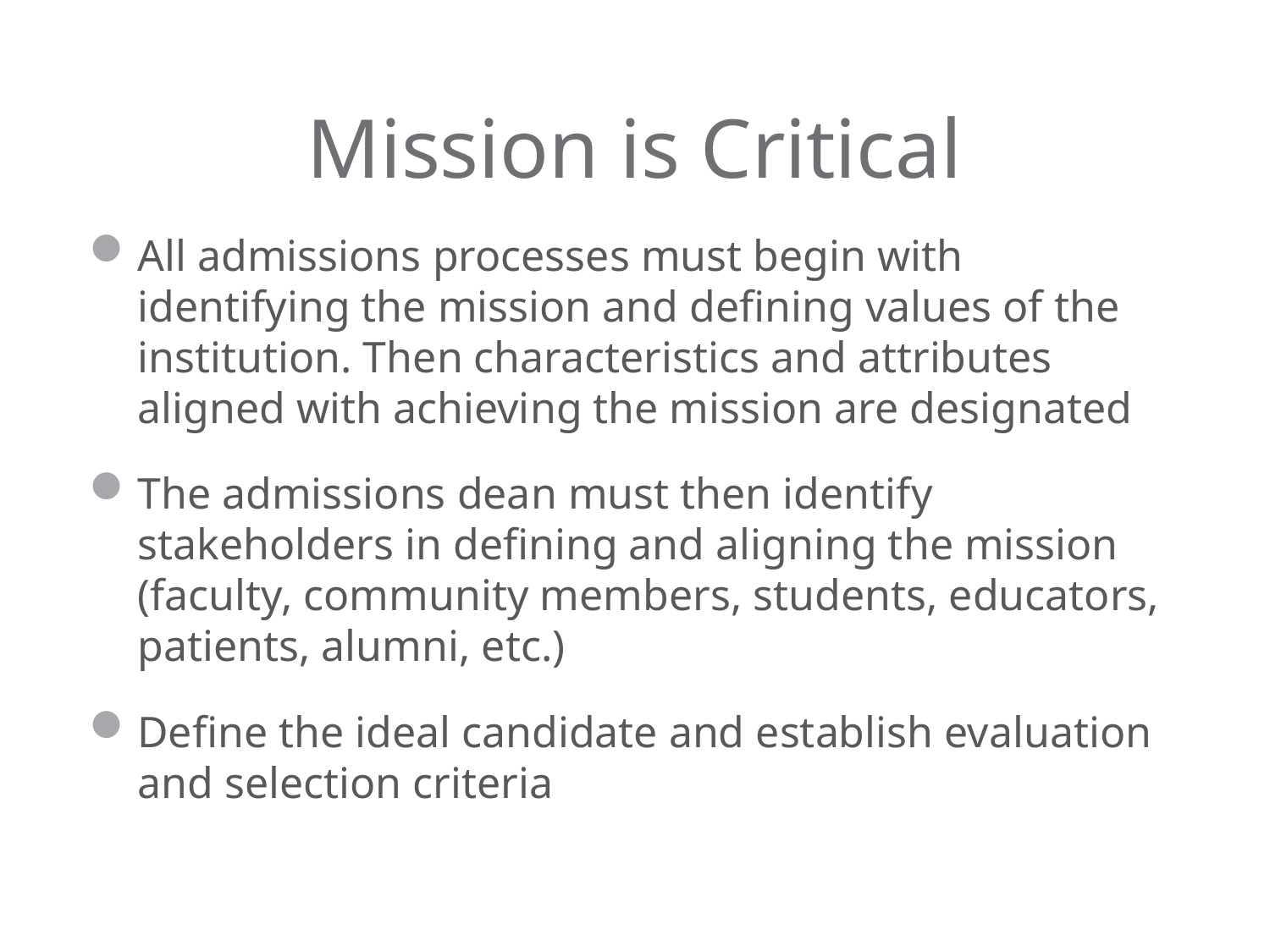

# Mission is Critical
All admissions processes must begin with identifying the mission and defining values of the institution. Then characteristics and attributes aligned with achieving the mission are designated
The admissions dean must then identify stakeholders in defining and aligning the mission (faculty, community members, students, educators, patients, alumni, etc.)
Define the ideal candidate and establish evaluation and selection criteria

## Slide 9
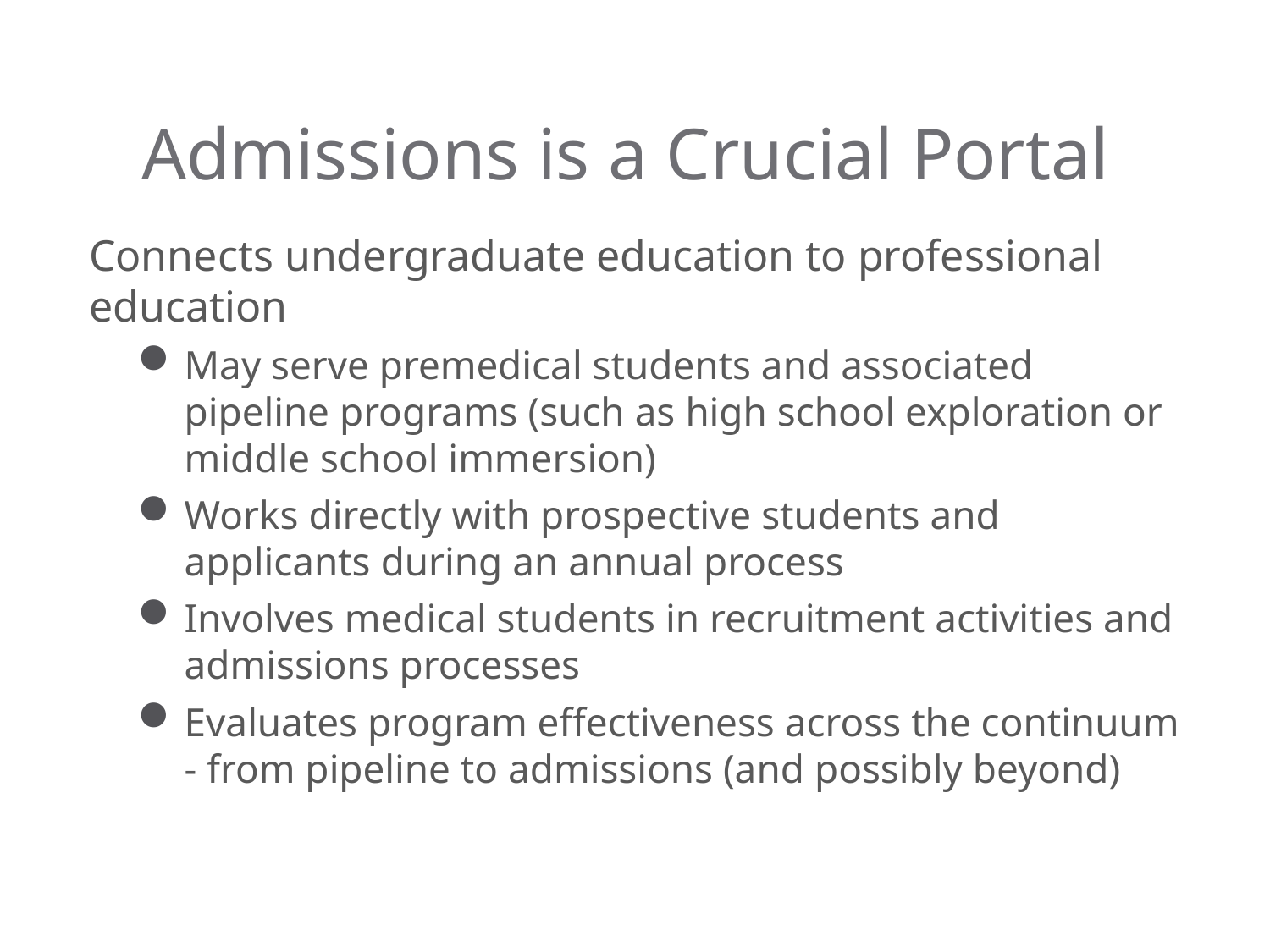

# Admissions is a Crucial Portal
Connects undergraduate education to professional education
May serve premedical students and associated pipeline programs (such as high school exploration or middle school immersion)
Works directly with prospective students and applicants during an annual process
Involves medical students in recruitment activities and admissions processes
Evaluates program effectiveness across the continuum - from pipeline to admissions (and possibly beyond)

## Slide 10
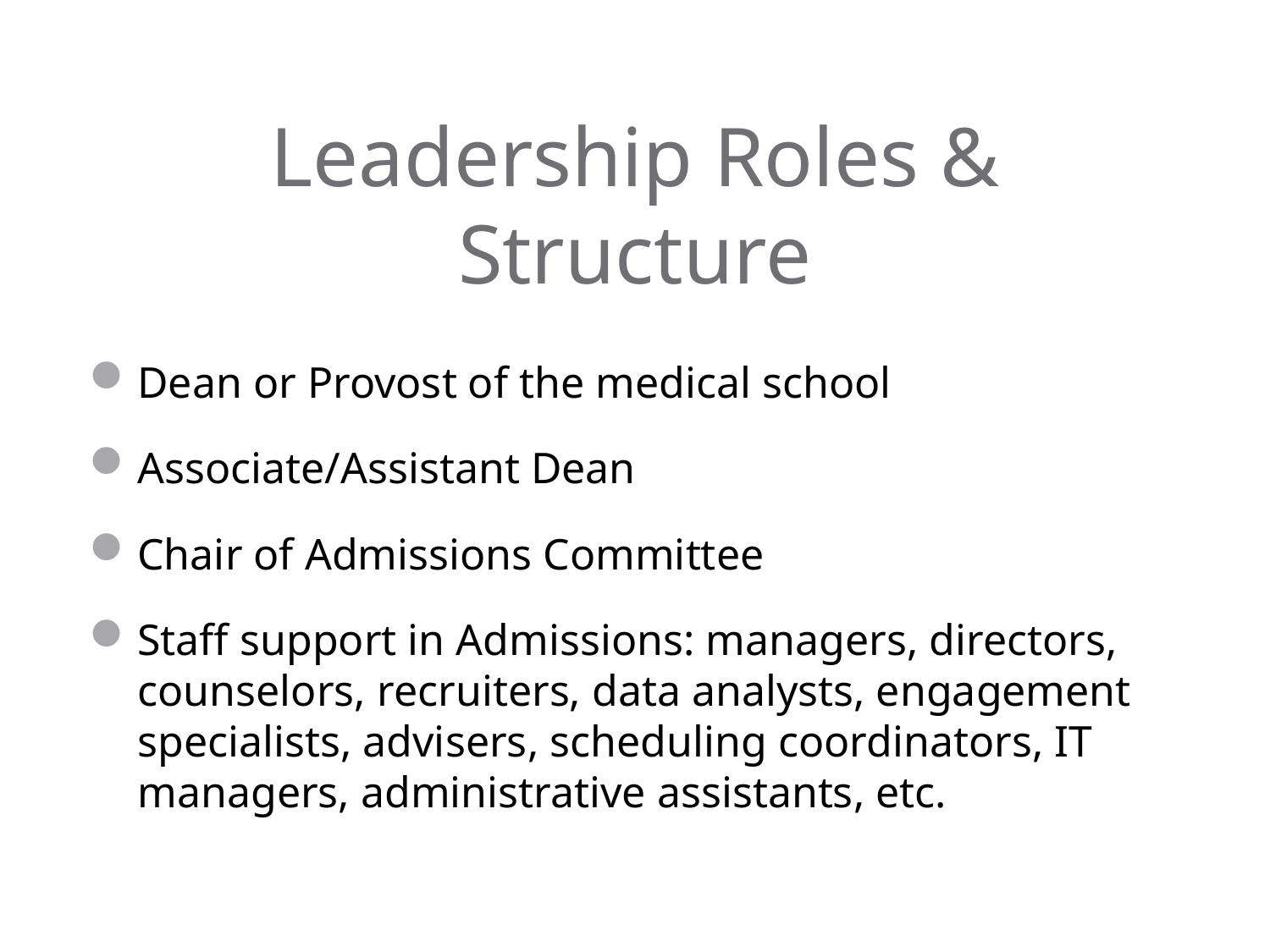

# Leadership Roles & Structure
Dean or Provost of the medical school
Associate/Assistant Dean
Chair of Admissions Committee
Staff support in Admissions: managers, directors, counselors, recruiters, data analysts, engagement specialists, advisers, scheduling coordinators, IT managers, administrative assistants, etc.

## Slide 11
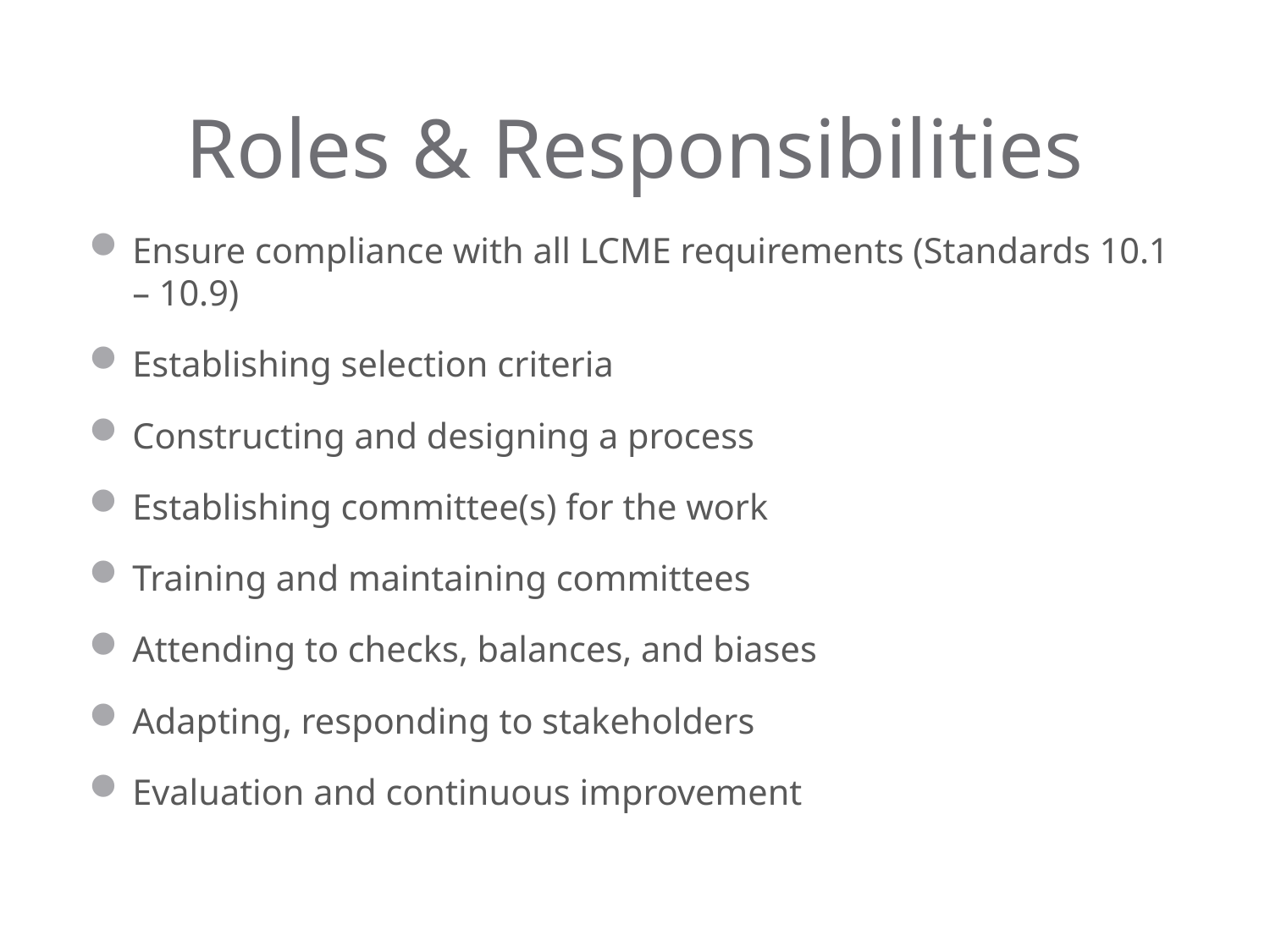

# Roles & Responsibilities
Ensure compliance with all LCME requirements (Standards 10.1 – 10.9)
Establishing selection criteria
Constructing and designing a process
Establishing committee(s) for the work
Training and maintaining committees
Attending to checks, balances, and biases
Adapting, responding to stakeholders
Evaluation and continuous improvement

## Slide 12
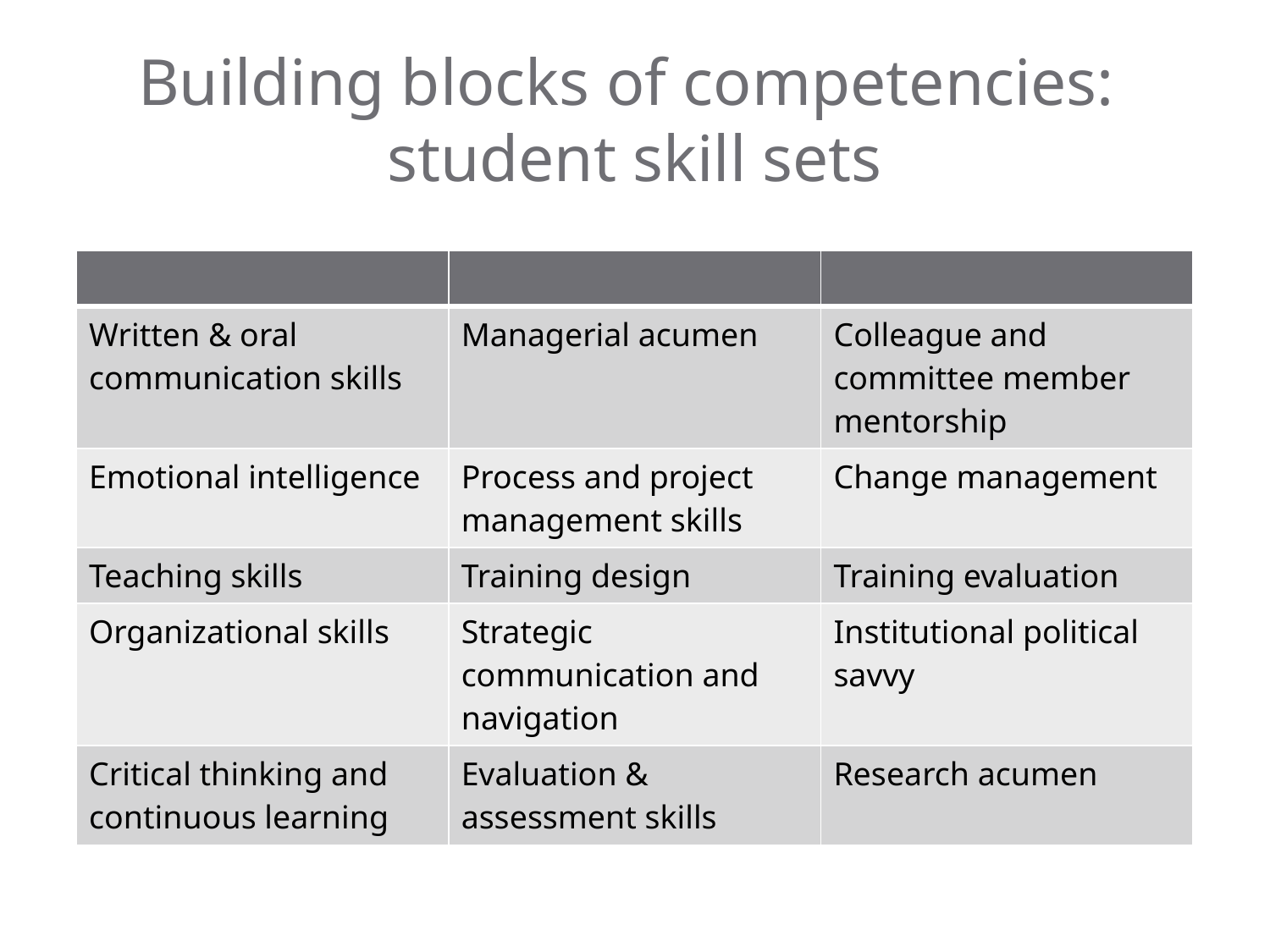

# Building blocks of competencies: student skill sets
| | | |
| --- | --- | --- |
| Written & oral communication skills | Managerial acumen | Colleague and committee member mentorship |
| Emotional intelligence | Process and project management skills | Change management |
| Teaching skills | Training design | Training evaluation |
| Organizational skills | Strategic communication and navigation | Institutional political savvy |
| Critical thinking and continuous learning | Evaluation & assessment skills | Research acumen |

## Slide 13
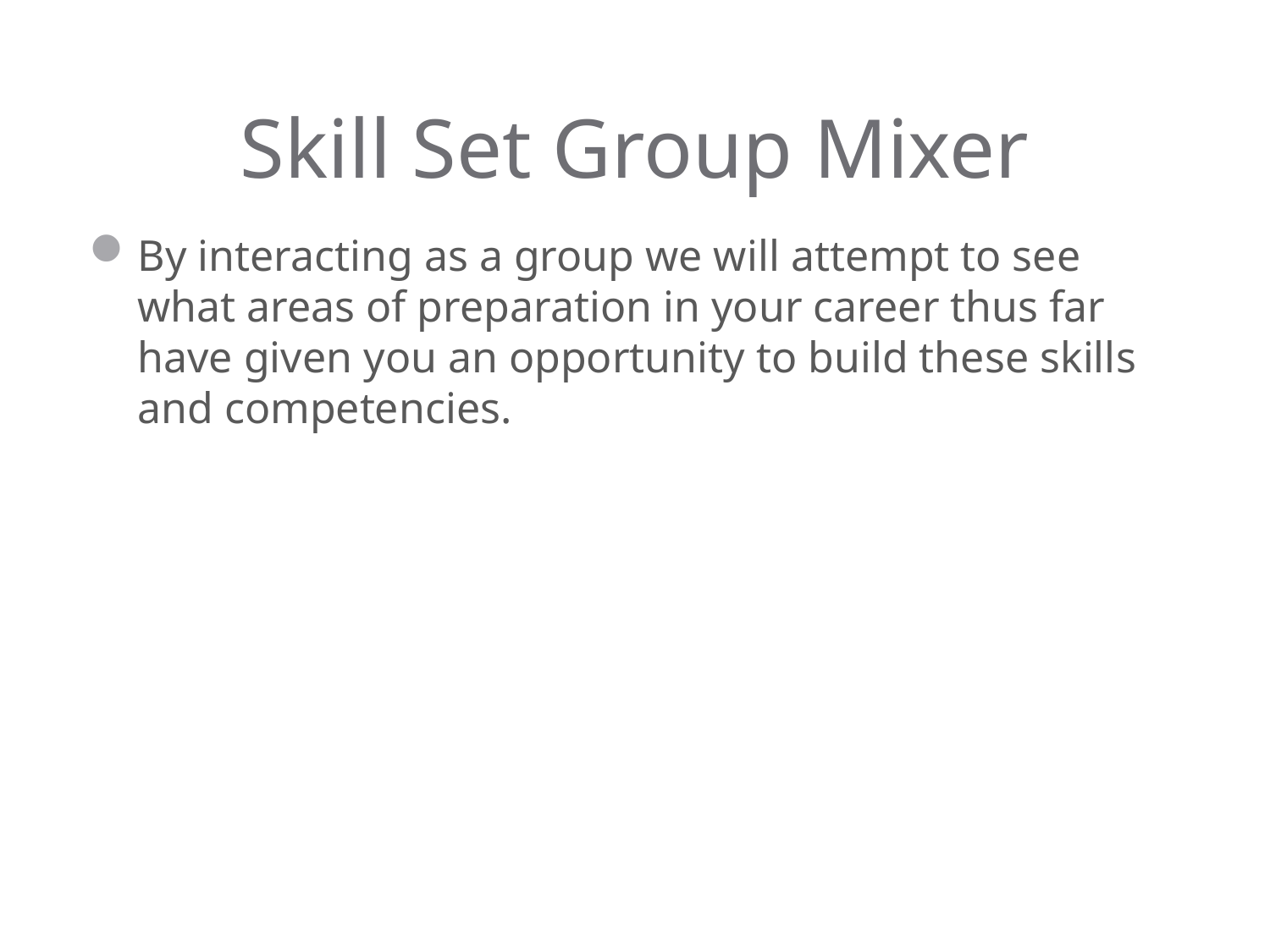

# Skill Set Group Mixer
By interacting as a group we will attempt to see what areas of preparation in your career thus far have given you an opportunity to build these skills and competencies.

## Slide 14
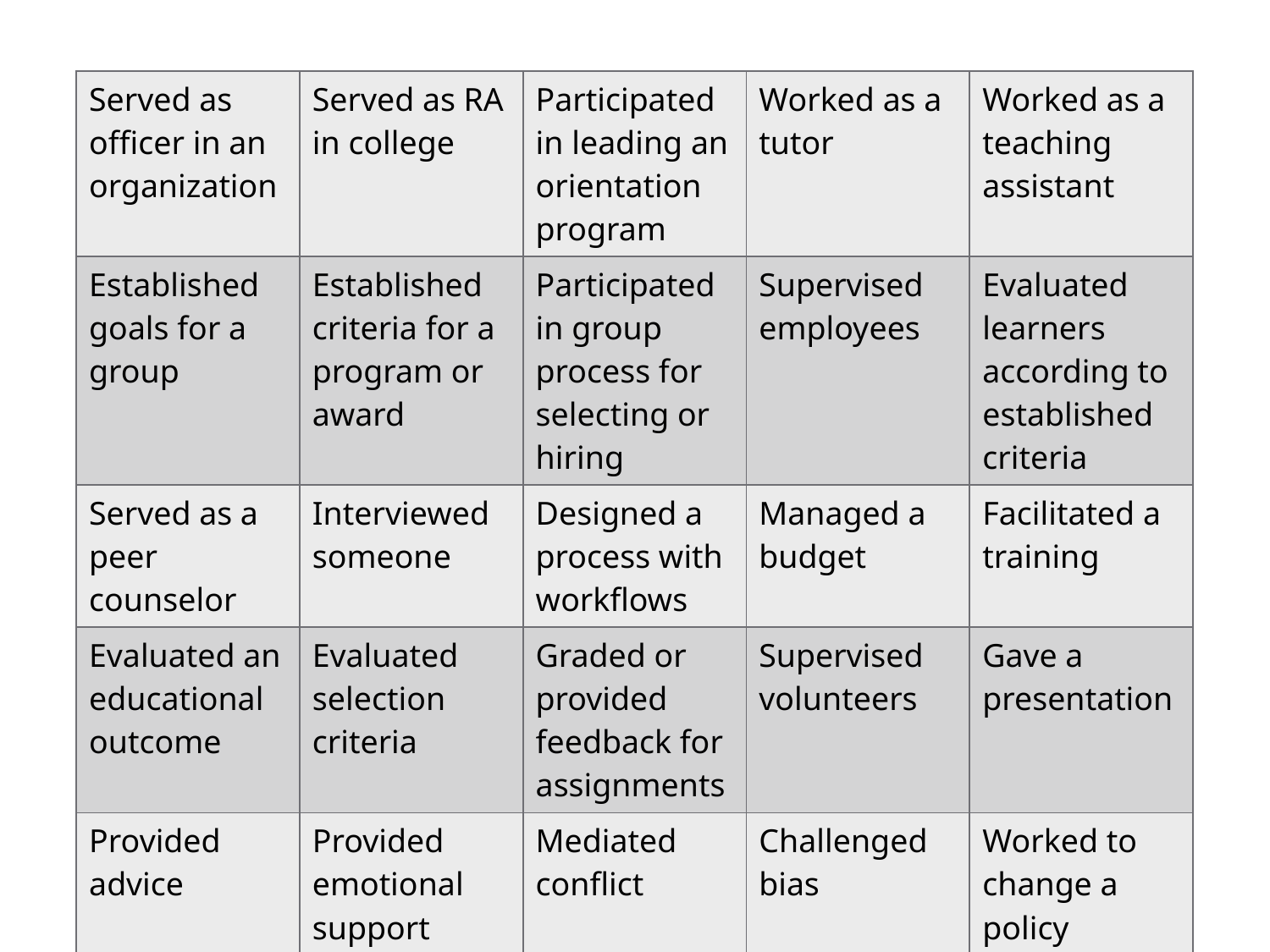

| Served as officer in an organization | Served as RA in college | Participated in leading an orientation program | Worked as a tutor | Worked as a teaching assistant |
| --- | --- | --- | --- | --- |
| Established goals for a group | Established criteria for a program or award | Participated in group process for selecting or hiring | Supervised employees | Evaluated learners according to established criteria |
| Served as a peer counselor | Interviewed someone | Designed a process with workflows | Managed a budget | Facilitated a training |
| Evaluated an educational outcome | Evaluated selection criteria | Graded or provided feedback for assignments | Supervised volunteers | Gave a presentation |
| Provided advice | Provided emotional support | Mediated conflict | Challenged bias | Worked to change a policy |

## Slide 15
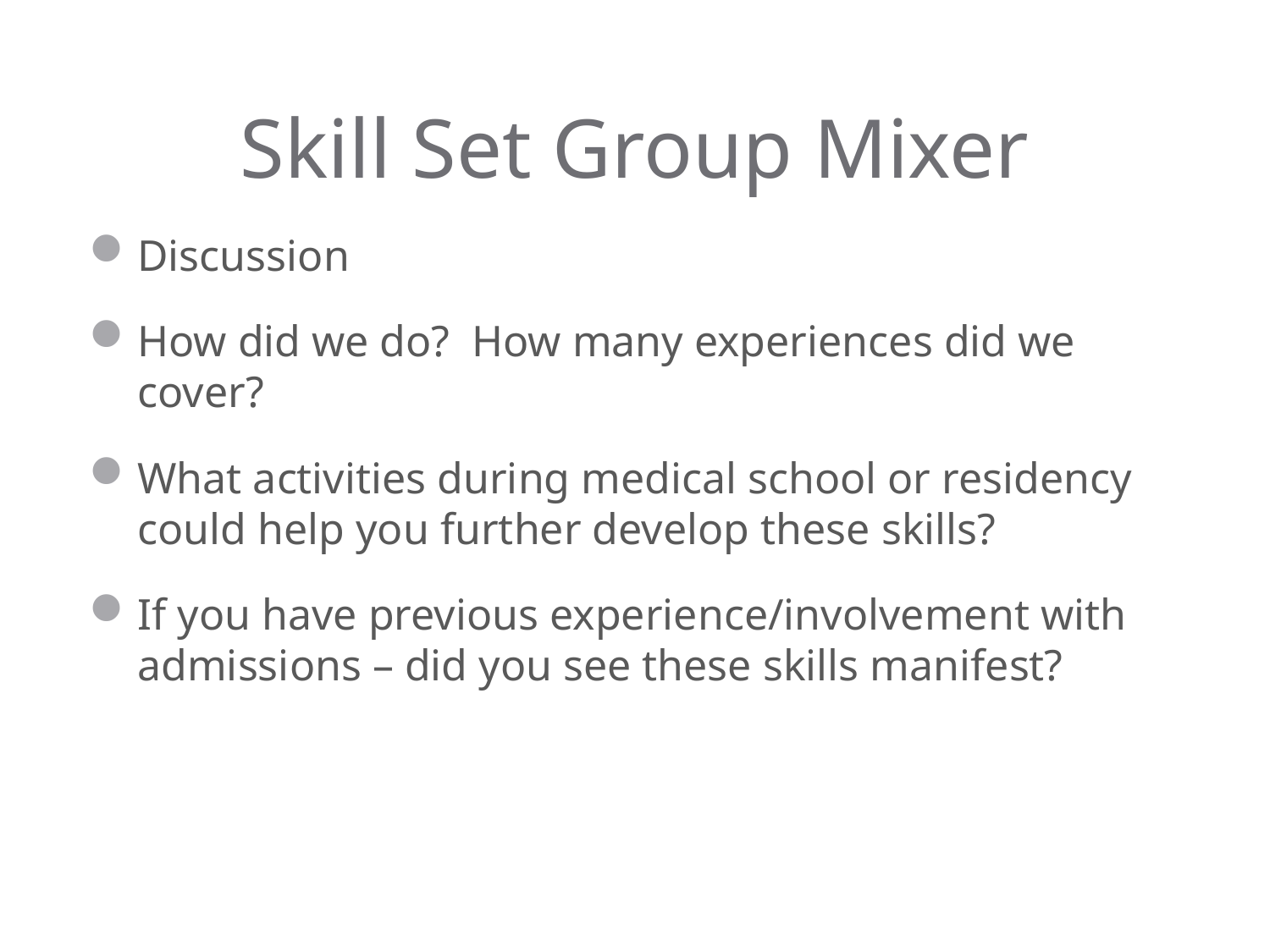

# Skill Set Group Mixer
Discussion
How did we do? How many experiences did we cover?
What activities during medical school or residency could help you further develop these skills?
If you have previous experience/involvement with admissions – did you see these skills manifest?

## Slide 16
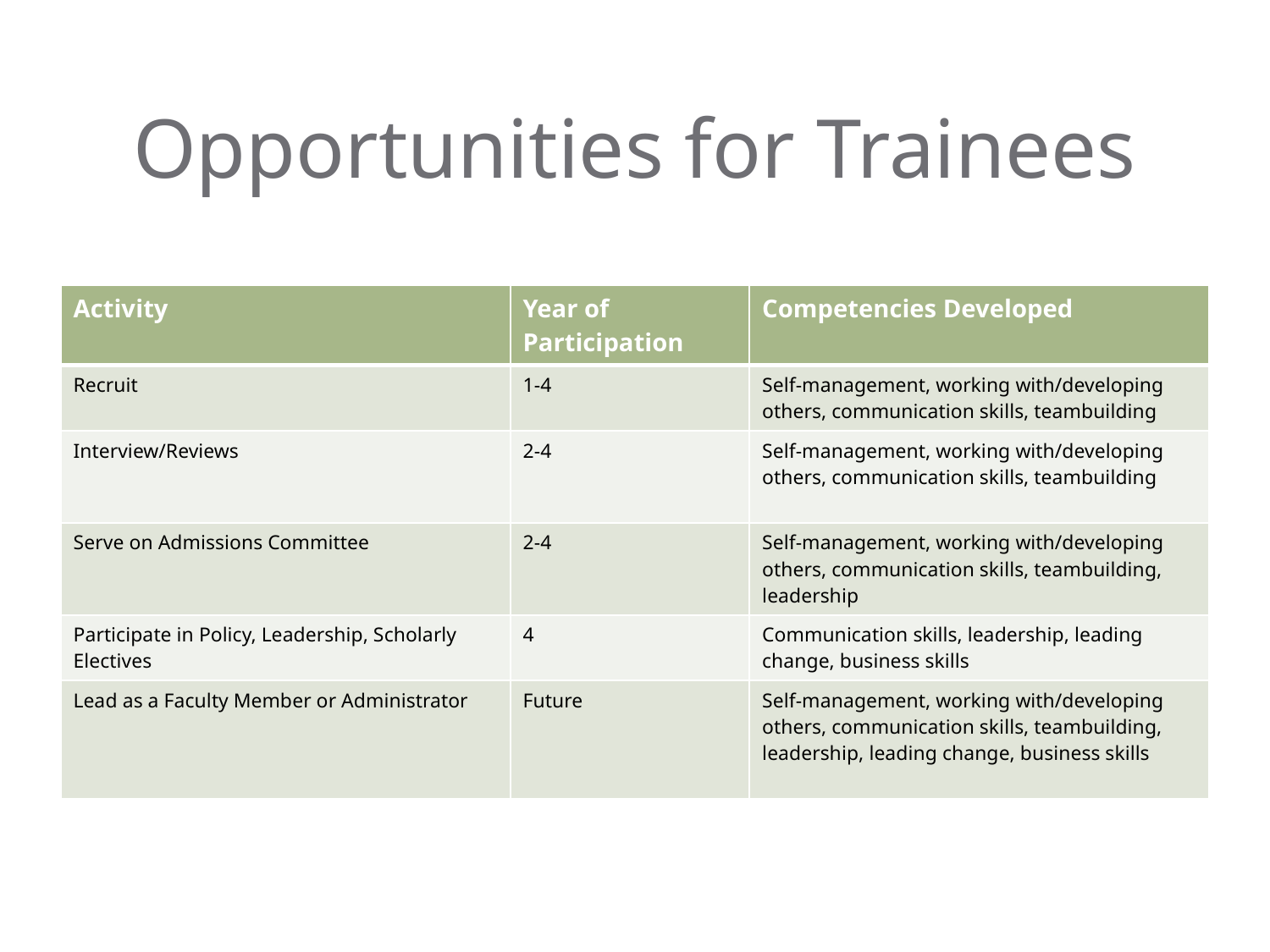

# Opportunities for Trainees
| Activity | Year of Participation | Competencies Developed |
| --- | --- | --- |
| Recruit | 1-4 | Self-management, working with/developing others, communication skills, teambuilding |
| Interview/Reviews | 2-4 | Self-management, working with/developing others, communication skills, teambuilding |
| Serve on Admissions Committee | 2-4 | Self-management, working with/developing others, communication skills, teambuilding, leadership |
| Participate in Policy, Leadership, Scholarly Electives | 4 | Communication skills, leadership, leading change, business skills |
| Lead as a Faculty Member or Administrator | Future | Self-management, working with/developing others, communication skills, teambuilding, leadership, leading change, business skills |

## Slide 17
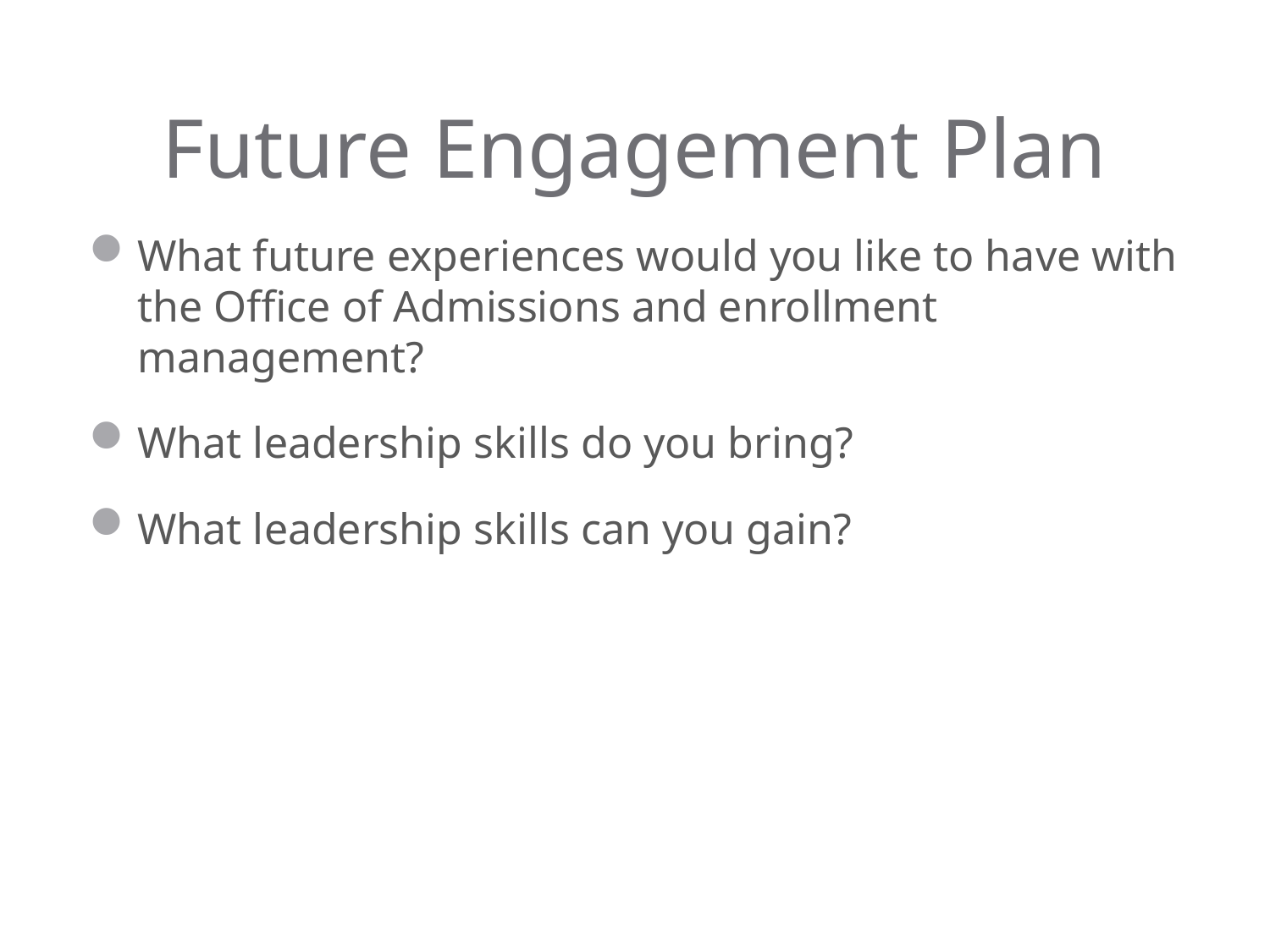

# Future Engagement Plan
What future experiences would you like to have with the Office of Admissions and enrollment management?
What leadership skills do you bring?
What leadership skills can you gain?

## Slide 18
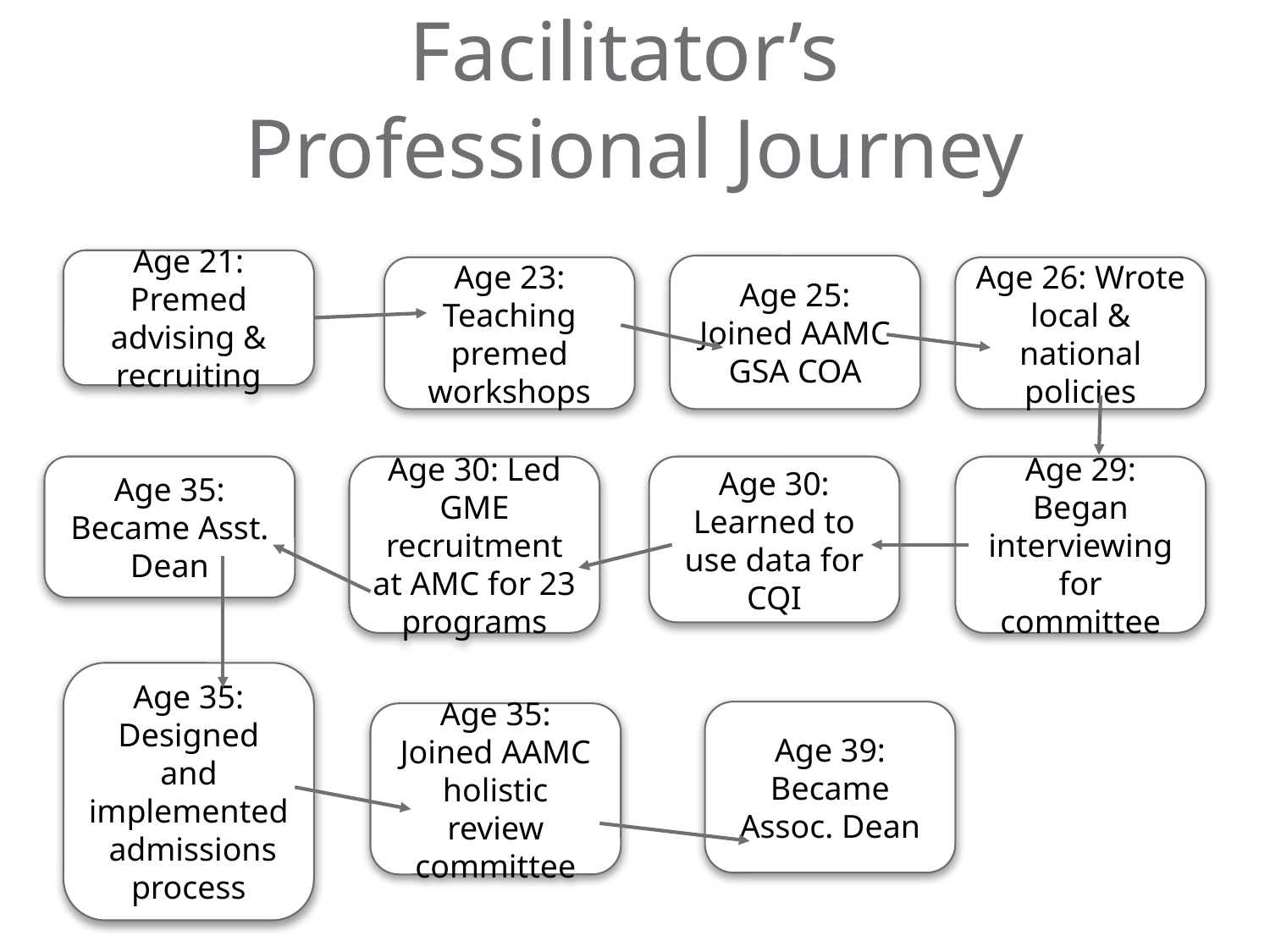

# Facilitator’s Professional Journey
Age 21: Premed advising & recruiting
Age 25: Joined AAMC GSA COA
Age 23: Teaching premed workshops
Age 26: Wrote local & national policies
Age 35: Became Asst. Dean
Age 30: Led GME recruitment at AMC for 23 programs
Age 30: Learned to use data for CQI
Age 29: Began interviewing for committee
Age 35: Designed and implemented admissions process
Age 39: Became Assoc. Dean
Age 35: Joined AAMC holistic review committee

## Slide 19
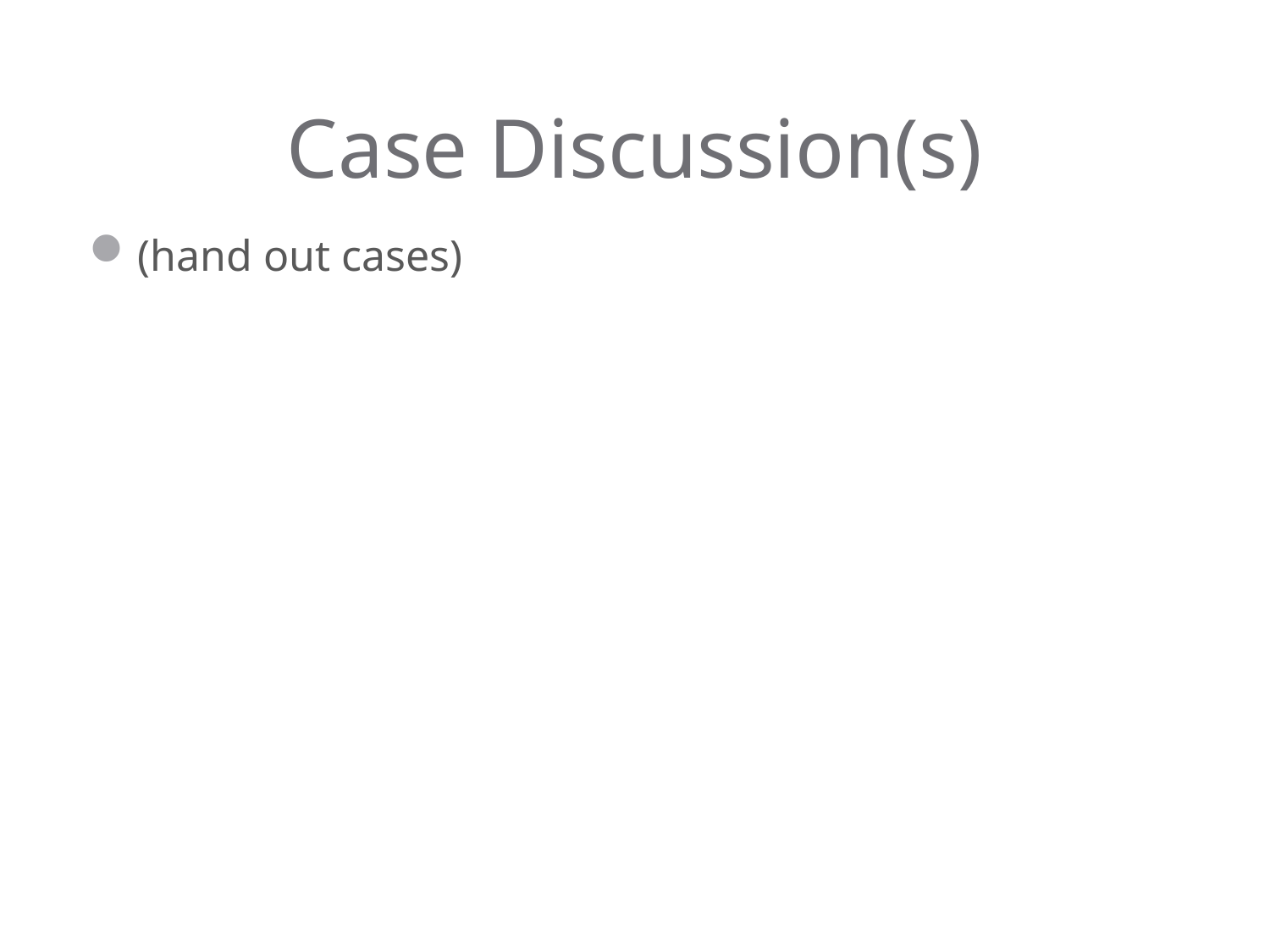

# Case Discussion(s)
(hand out cases)

## Slide 20
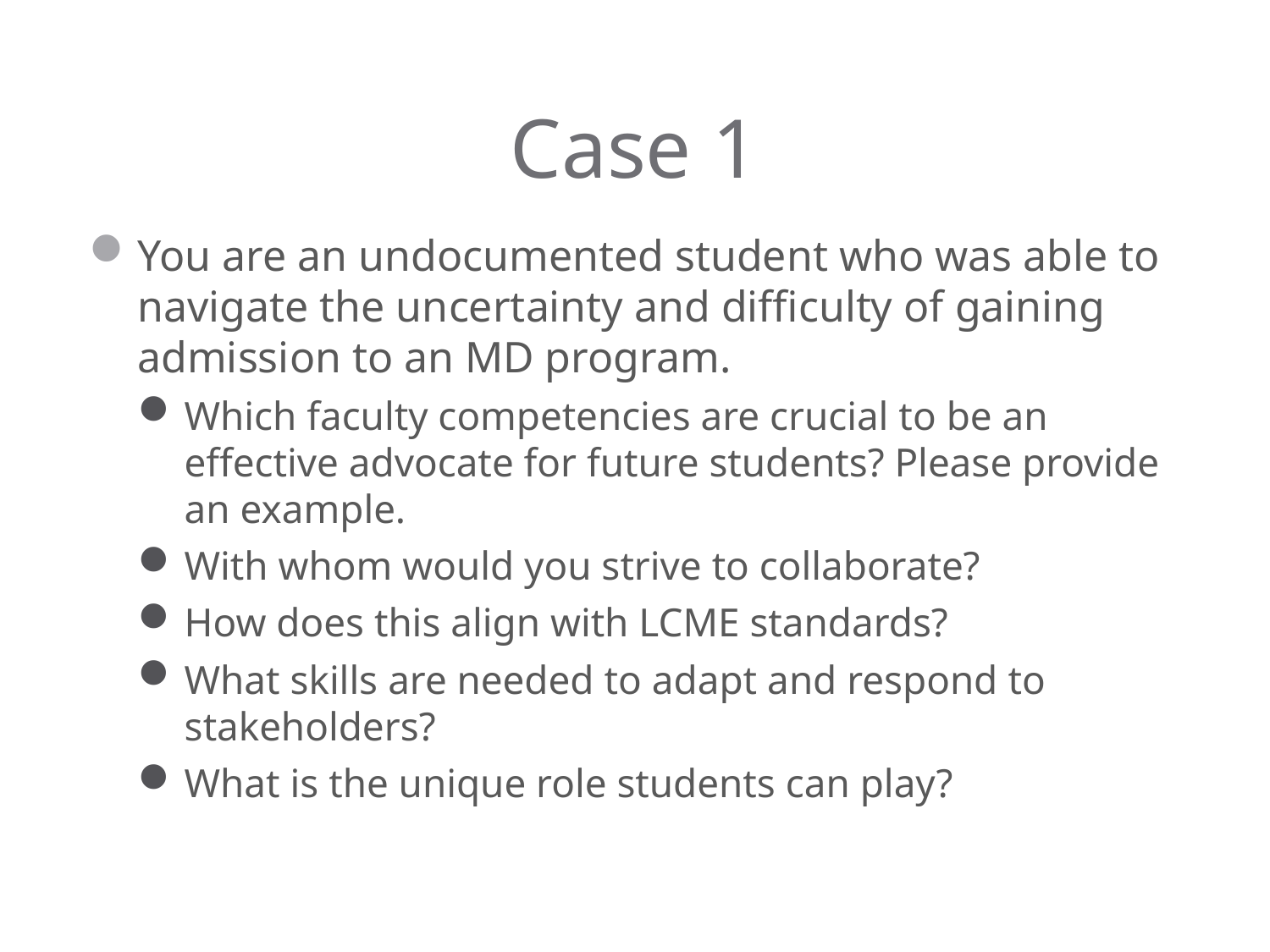

# Case 1
You are an undocumented student who was able to navigate the uncertainty and difficulty of gaining admission to an MD program.
Which faculty competencies are crucial to be an effective advocate for future students? Please provide an example.
With whom would you strive to collaborate?
How does this align with LCME standards?
What skills are needed to adapt and respond to stakeholders?
What is the unique role students can play?

## Slide 21
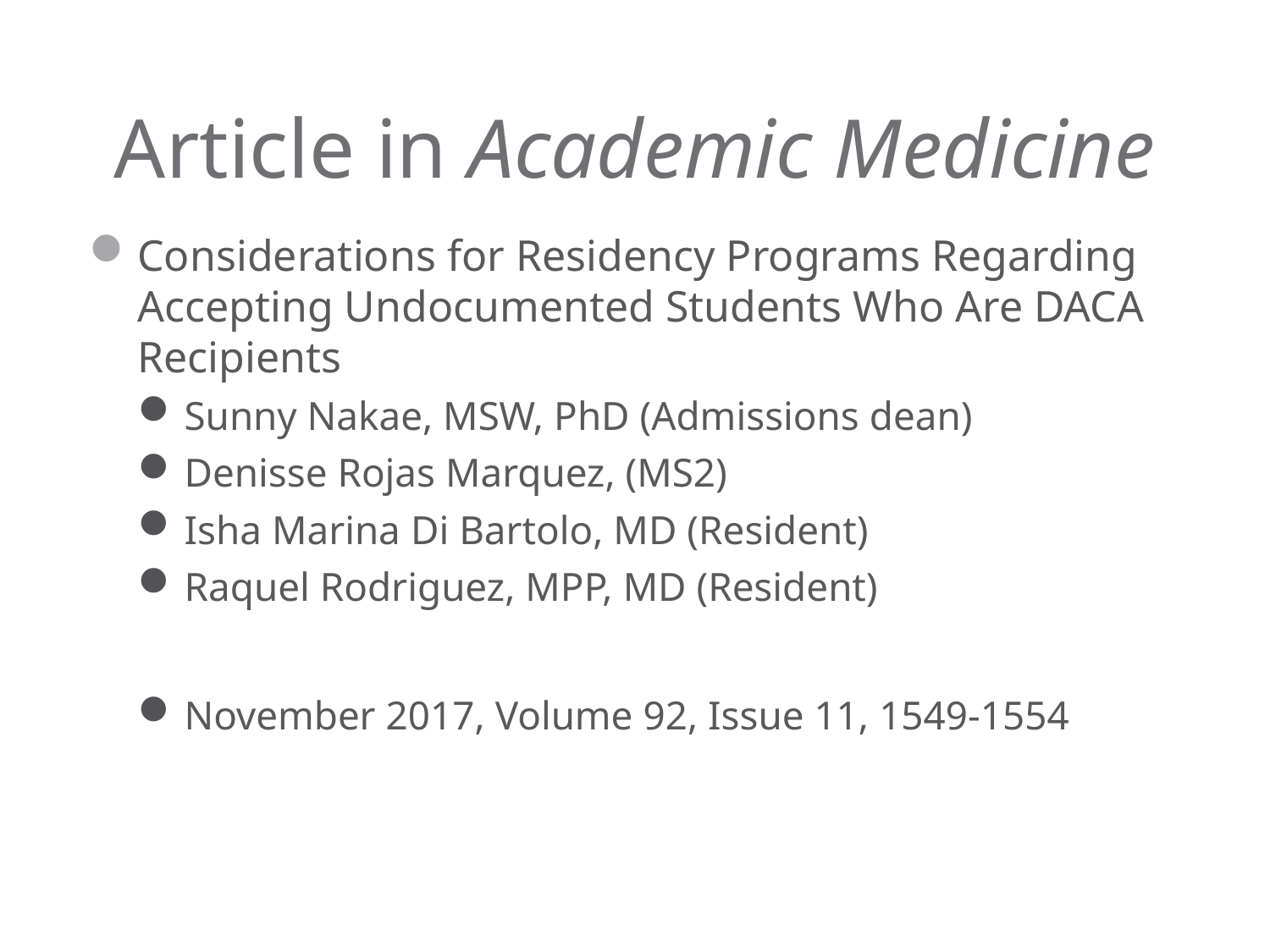

# Article in Academic Medicine
Considerations for Residency Programs Regarding Accepting Undocumented Students Who Are DACA Recipients
Sunny Nakae, MSW, PhD (Admissions dean)
Denisse Rojas Marquez, (MS2)
Isha Marina Di Bartolo, MD (Resident)
Raquel Rodriguez, MPP, MD (Resident)
November 2017, Volume 92, Issue 11, 1549-1554

## Slide 22
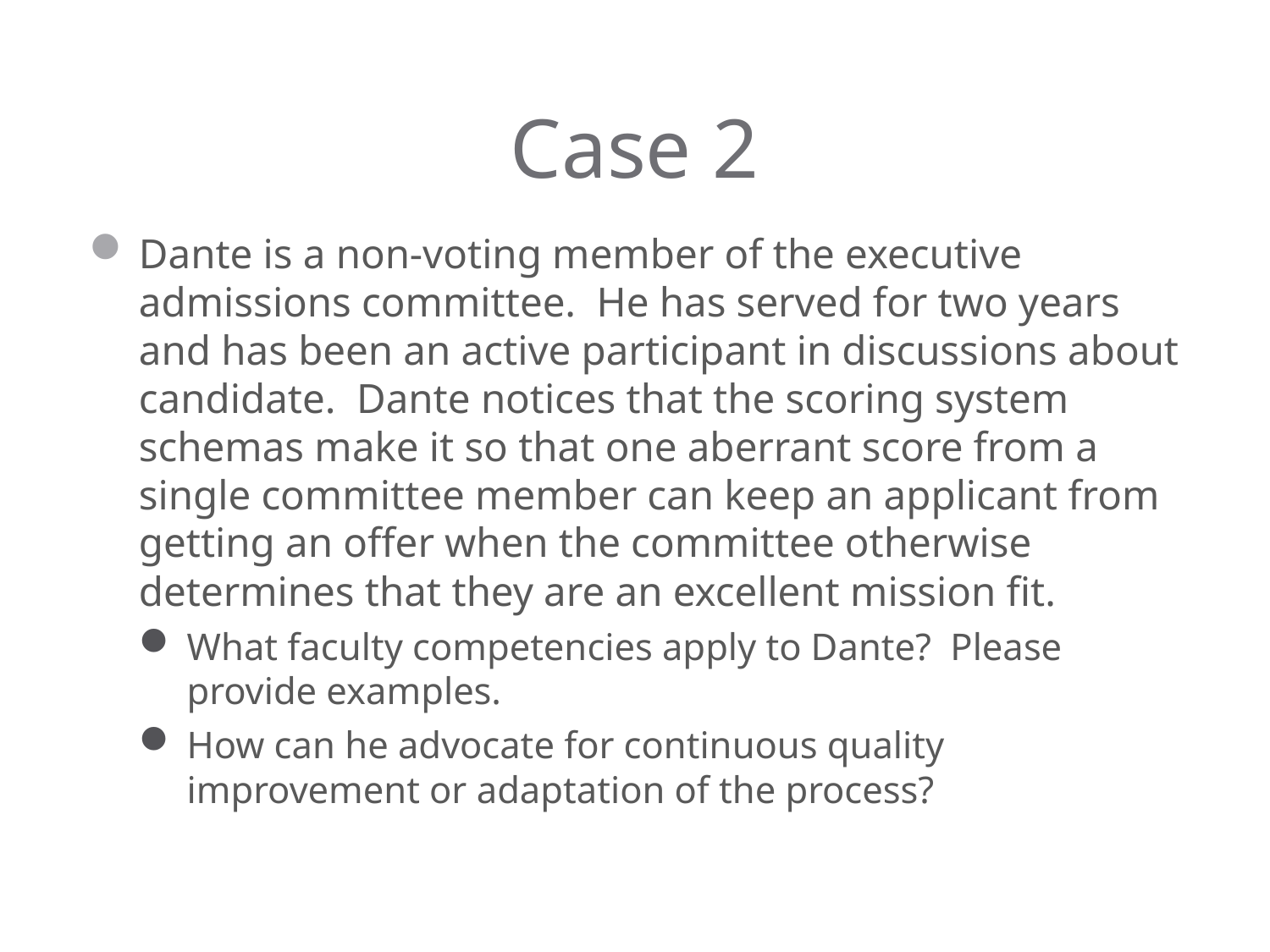

# Case 2
Dante is a non-voting member of the executive admissions committee. He has served for two years and has been an active participant in discussions about candidate. Dante notices that the scoring system schemas make it so that one aberrant score from a single committee member can keep an applicant from getting an offer when the committee otherwise determines that they are an excellent mission fit.
What faculty competencies apply to Dante? Please provide examples.
How can he advocate for continuous quality improvement or adaptation of the process?

## Slide 23
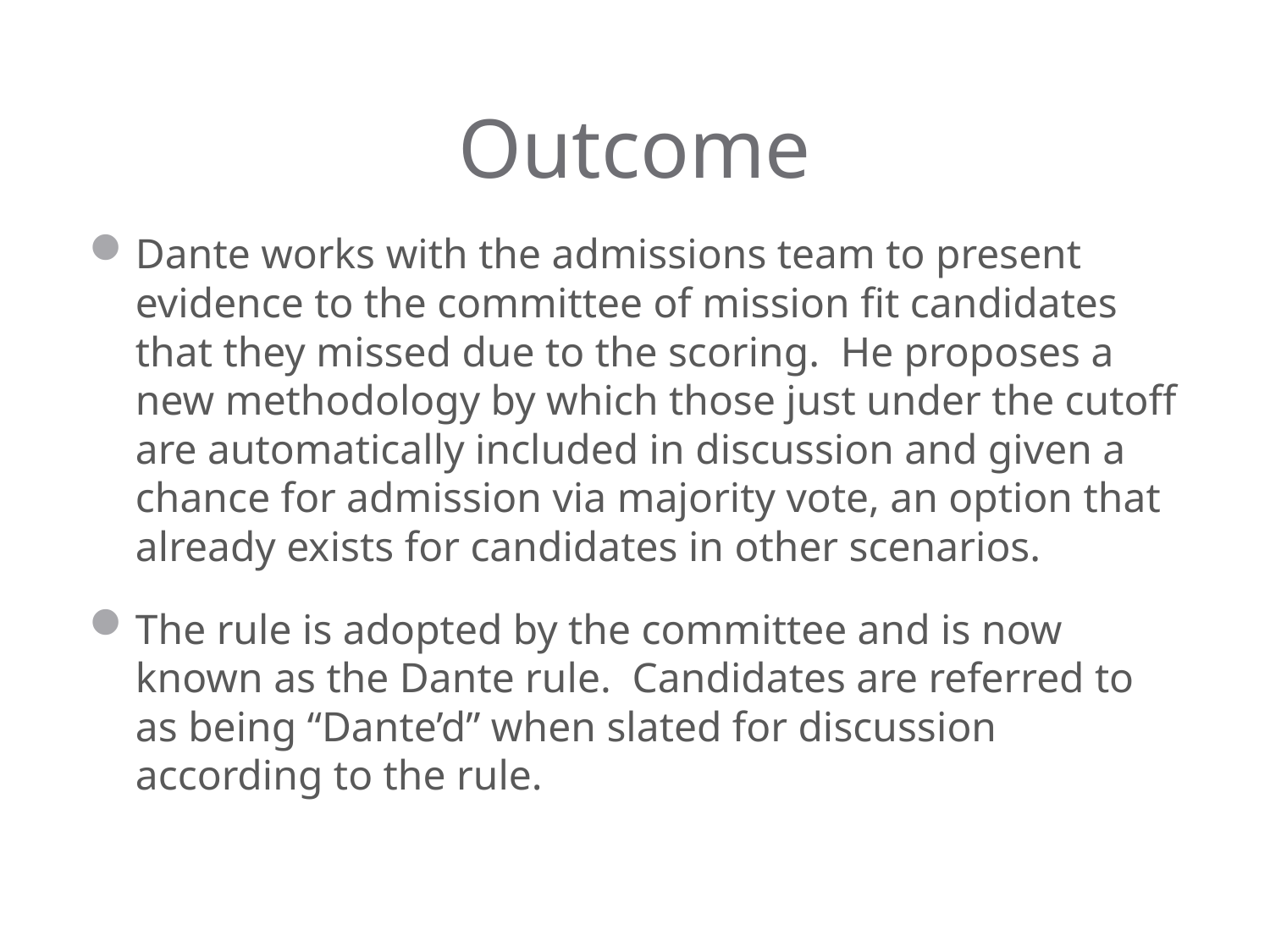

# Outcome
Dante works with the admissions team to present evidence to the committee of mission fit candidates that they missed due to the scoring. He proposes a new methodology by which those just under the cutoff are automatically included in discussion and given a chance for admission via majority vote, an option that already exists for candidates in other scenarios.
The rule is adopted by the committee and is now known as the Dante rule. Candidates are referred to as being “Dante’d” when slated for discussion according to the rule.

## Slide 24
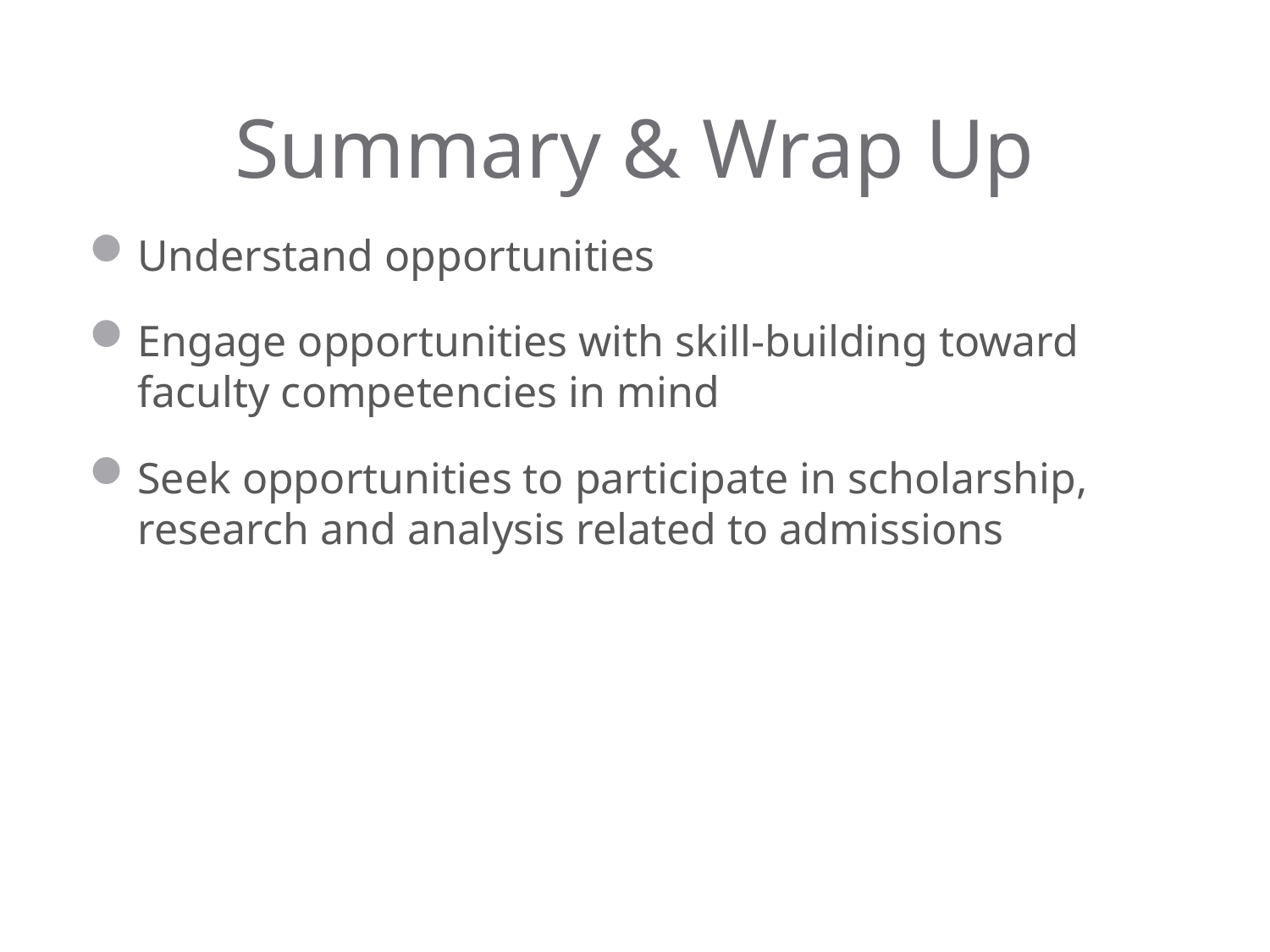

# Summary & Wrap Up
Understand opportunities
Engage opportunities with skill-building toward faculty competencies in mind
Seek opportunities to participate in scholarship, research and analysis related to admissions

## Slide 25
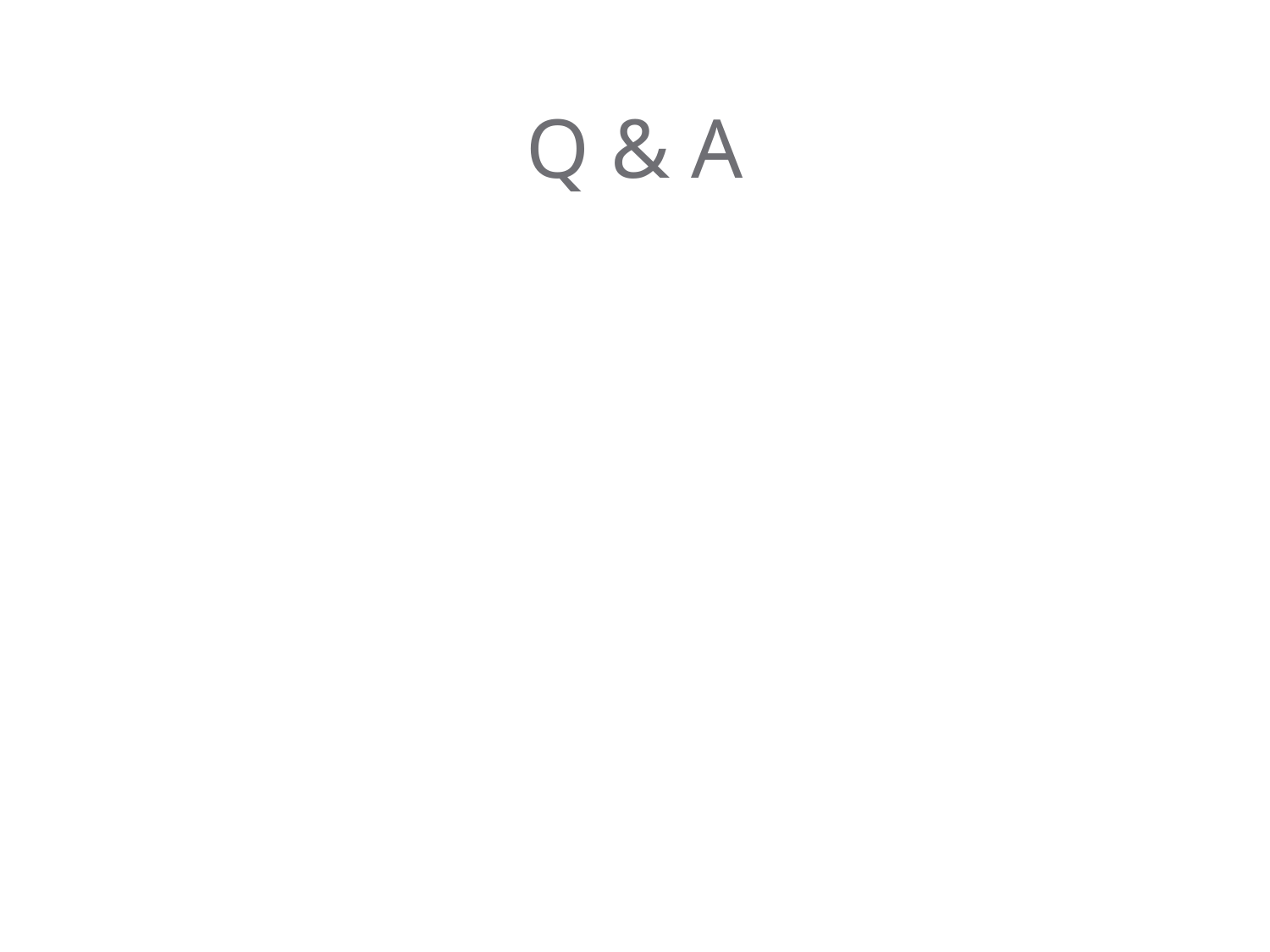

# Q & A
