# Supplementary material for: Office of Admissions: Engagement and Leadership Opportunities for Trainees
Source: MedEdPORTAL. 2020 Nov 24;16:11018. doi: 10.15766/mep_2374-8265.11018 (PMC7703483; doi:10.15766/mep_2374-8265.11018)
Supplement: Supplementary file 1 — PowerPoint Presentation.pptxFacilitator Guide.docxPrereading Assignment.docxSkill-Set Group Mixer.docxAdmission Cases.docxPre- and Postworkshop Survey.docx [file mep_2374-8265.11018-s001.zip › B. Facilitator Guide.docx]

**Office of Admissions: Engagement and Leadership Opportunities for Trainees**

**Facilitator Instructional Guide**

**Overall Goals**

The goals of our workshop are to provide an understanding of engagement and leadership opportunities in admissions in medical education. For attendees that aspire to a career in academic medicine, this workshop will explain the skill sets and opportunities relevant to this leadership pathway. This workshop may have 1-2 facilitators.

**Workshop Objectives**

- 1. Describe the functions of the Office of Admissions
  2. Describe leadership competencies that are associated with various roles within the Office of Admissions
  3. Identify opportunities for trainees to engage in the Office of Admissions
  4. Review examples of how trainees can facilitate change

**Workshop Handouts and Materials**

1. Appendix C- BNGAP Admissions Sample Job Description (pre-reading)
2. Appendix D- BNGAP Admissions Leadership Experiences Mixer
3. Appendix E- BNGAP Admissions Case Discussion Handout
4. Appendix F- Pre and post-workshop survey

**Suggested Agenda and Timeline**

- Pre-workshop evaluation. (3 minutes)
- Slide 1-13: 20 minutes
- Slides 14-16: Group activity and discussion, 15 minutes
- Slide 17-19: 10 minutes
- Slides 20-25: Case discussions, 12 minutes
- Slide 26: Questions and answers: 3 minutes
- Post-workshop evaluation. (3 minutes)

**Slide Instructions**

**Slide 1:**

Title slide: Office of Admissions: Engagement and Leadership Opportunities for Trainees

The facilitators should introduce themselves to the audience and discuss their roles in their respective institutions. The audience should be informed that the session will cover admissions using a macro lens. The frameworks discussed have the potential to evoke tangents and details related to individual institutional experiences, which this overview module does not allocate time for. It is advisable if using this as part of a series to revisit the topics or leadership areas in greater detail with subsequent sessions. The goal is to raise awareness among participants and give them insight into leadership in this critical area of academic medicine.

**Slide 2:**

Learning Objectives. Facilitator should state objectives and time line for the workshop and emphasize the following learning objectives:

- Describe the functions of the Office of Admissions
- Describe leadership competencies that are associated with various roles within the Office of Admissions
- Identify opportunities for trainees to engage in the Office of Admissions
- Review examples of how trainees can facilitate change s workshop will give an overview of medical school admissions.

**Slide 3 – Admissions Definition**

Provide the concise definition of admissions, add your aspects from your experience, but do not change the slide. See Group on Student Affairs Handbook for Admissions Officers for more detail (optional): <https://www.aamc.org/members/gsa/committees_gsa/coa/handbook/>

**Slide 4 – Opening Reflection:**

Pose these questions to the attendees to have a brief open reflection to give facilitators a sense of the experiences among the group. For 60 minute workshops, limit discussion to 3 minutes. If you are allocating more time, adjust accordingly.

**Slide 5 Reflection Part II:**

Refer briefly to the handout for Admissions Dean Sample Job Description (pre-reading). Segue the tasks and job description into leadership components necessary for the role.

**Slide 6 – Faculty Leadership Competencies:**

Here are the main leadership competencies that trainees can develop and master through engagement in admissions.

Faculty Leadership Competencies Achievable Through Admissions-Related Activities (see: Lucas R, Goldman EF., Scott AR et al. Leadership Development Programs at Academic Health Centers: Results of a National Survey. Academic Medicine. 93(2):229-236, February 2018.)

**Self-management:** demonstrates emotional intelligence in encounters with interviewees in a judicious and empathetic manner

**Working with and developing others:**  coaches peers in serving as effective tour guides, interviewers and committee members

**Leading change:** understands change management through application of new or revised policies and procedures

**Communication skills**: creates a welcoming space to effectively convey information and listen to applicants

**Teambuilding**: works with fellow admissions staff to problem solve and make decisions about applicants

**Leadership:** understands the structure and culture of the Office of Admissions

**Business skills:** evaluates and improves admissions policies and procedures through quality improvement efforts

It is important for students who engage in admissions related work to not only reflect on the competencies listed above but also use the terms in describing their efforts on their CV, ERAS or fellowship applications, or other promotion documents. Such use conveys to a reviewer the applicants critical thought and understanding of the benefits of engagement in admissions work.

**Slide 7 – Admissions Overview:**

Transition slide to components of admissions in medicine

**Slide 8 – Mission is Critical:**

Emphasize mission as a key anchor to any admissions process. Every institution must define and codify what mission means and how it is evaluated in candidates. See Holistic Review Initiative (optional): <https://www.aamc.org/initiatives/holisticreview/>

**Slide 9 – Admissions is a Crucial Portal:**

Describe ways that admission serves as a portal that bridges premed activities to professional education. [Use a photo of your own pipeline programs in action, or your pipeline website and remove text if you wish.]

Admissions connects undergraduate education to professional education:

- May serve premedical students and associated pipeline programs (such as high school exploration or middle school immersion)
- Works directly with prospective students and applicants during an annual process
- Involves medical students in recruitment activities and admissions processes
- Evaluates program effectiveness across the continuum - from pipeline to admissions (and possibly beyond)

**Slide 10 – Leadership Roles and Structures:**

Describe the admissions leadership team at your institution and the associated support staff from ancillary areas. Include a photo of your team if you wish [replace slide text.]

- Dean or Provost of the medical school
- Associate/Assistant Dean
- Chair of Admissions Committee
- Staff support in Admissions: managers, directors, counselors, recruiters, data analysts, engagement specialists, advisers, scheduling coordinators, IT managers, administrative assistants, etc.

See LCME standards, 10.2, and 10.3. Note to learners that student preparation and pipeline programs may also be within the purview of the admissions office - directly or indirectly. The class is selected from students that apply to the school, so pipeline programs are essential in ensuring a diverse applicant pool. This is LCME standard 3.3.

**Slide 11 – Roles and Responsibilities:**

Describe the roles of admissions and emphasize the specific LCME standards 10.1-10.9.

- Selection criteria:
  - What is our vision for our graduates and how do those qualities translate into preparation areas? (goes back to mission!)
  - What external factors need to be met to create a process (state vs. local, public vs. private, undergraduate guarantee programs, etc.)
  - Criteria must be applied uniformly and equitably across the pool. Schools can establish any reasonable guidelines, but those guidelines must be equitably followed.
- Constructing and designing a process:
  - Recruiting
  - Evaluating/reviewing/screening
  - Conducting in-person interview(s)
  - Establishing parameters, policies and procedures for committee decisions
  - Managing rules, regulations and policies in a local and national context
- Establishing committees for the work:
  - Identifying members and recruiting for committees
  - How will each phase be implemented?
  - What committee structure works best for your institution and its demands?
  - What kind of diversity is necessary to successfully implement the process? (basic science and MD mix, students, staff, community members, alums, paid screeners, etc.)
  - Establishing regular communication with committee members
- Training committees:
  - Training committee members with essential elements for the job
  - Utilizing technology in savvy ways to maximize efficiency
  - Ensuring proper onboarding with proper confidentiality and conflict of interest notations.
  - Understanding principles of enrollment management and holistic review
  - Ongoing training to provide feedback to committee members
- Checks and balances:
  - Ethical considerations
  - Shared decision making – mitigating the influence of singular committee members
  - Managing biases personally and structurally
  - Identifying and managing conflicts of interest in advance
  - Maintaining adherence to privacy practices and legal concerns
- Adapting and responding:
  - Legal frameworks and decisions that impact policy
  - National tools, trends and changes in application components, evaluation tools, or common/recommended practices
  - Communicating potential impacts and changes to constituents – utilizing committee leadership to assist in managing change where appropriate
  - Engaging in continuous quality improvement
- Evaluation and improvement:
  - Closing the loop with UME colleagues. Tracking outcomes related to performance and mission adherence
  - Evaluating the process for points of improvement – rater agreement, instrument consistency, outcomes data, etc.
  - Communicating with and managing committees long term – succession plan, term limits, policies and procedures, annual training, on-boarding processes, addressing problems or inconsistencies
  - Providing feedback to the committees annually
  - Expressing appreciation for efforts
  - Continuing to cultivate skill sets among the committee members for specific roles
  - Providing opportunities for students to participate and learn
  - Engaging stakeholders regularly

**Slide 12:**

Transition to activity for skill set bingo. Provide these examples of skills sets that are important for admissions activities and leadership.

**Slide 13 – Skill Set Group Mixer:**

Let’s do a group activity to identify the skills and experiences among the group that apply to leadership within Admissions. 10 minutes.

Print Appendix BNGAP Admissions Leadership Experiences Group Mixer so there is one copy per attendee. Each attendee also needs a pen or pencil.

Ask attendees to mingle and find individuals in the room who have experience in one of the areas on your leadership skills mingle sheet. Participants will sign the box where they have the experience. For example, if a student has helped to change a policy during undergrad or med school, they would sign their name in that box. Ask participants to fill their sheet as quickly as possible. Limit duplicate signatures according to group size. There are 25 boxes. For example, if there are 16 participants, limit each participant to signing three boxes on any one participant’s sheet to encourage mixing.

When time is up, reconvene the group.

**Slide 14 – Mixer Visual**

This is a slide that shows the handout of the mixer grid that participants have. Leave it up during the activity.

**Slide 15 – Skills Set Mixer Discussion:**

Discuss skill set mixer using the prompts provided. 5 minutes.

- How did we do? How many experiences did we cover?
- What activities during medical school or residency could help you further develop these skills?
- If you have previous experience/involvement with admissions – did you see these skills manifest?

**Slides 16 – Opportunities for trainees:**

Chart that includes opportunities for trainees from MS1 to faculty future.

Discuss each area of admissions from recruitment activities to interviewing and serving on selection committees. Point out the unique roles and contributions for student engagement. Emphasize the skill development and leadership competency for each area.

**Slide 17 – Future Engagement Plan:**

Attendees should be prompted to think about creating a plan for future engagement in admissions activities to further develop themselves as leaders. For sessions adapted to be longer, pause for discussion so that applicants may share their plans with the group. For a 60 minute session this is personal takeaway for participants.

**Slide 18 – Facilitator’s Professional Journey:**

Time permitted, this is an opportunity for facilitators to talk about one or more experiences in their career journey and how that situated them in their current role. You might create a timeline that shows the activities and ages you engaged in that led you to your current role. This slide contains an example.

**Slide 19 – Case discussions:**

Transition to cases. Use Cases handout; one per participant. Break into small groups if needed.

**Slide 20 – Case 1 Undocumented Students:**

- Which faculty competencies are crucial to be an effective advocate for future students?
  - Leading change
  - Communication skills
  - Leadership
  - Working and developing others
- With whom would you strive to collaborate?
  - Senior leadership, financial aid experts, legal experts, classmates who are undocumented students or allies, community members who are undocumented or allies, foundations or non-profits engaging in advocacy, large expert institutions like AAMC, ACGME, AMA, etc.
- How does this align with LCME standards?
  - 10.3 – Policies regarding student selection/progress and their dissemination, 10.4 – characteristics of accepted applicants, 10.6 – content of informational materials
- What skills are needed to adapt and respond to stakeholders?
  - Leading change
  - Communication skills
  - Leadership
  - Working and developing others
  - Business acumen for the financial aid aspects
- What is the unique role students can play?
  - Bringing experiential expertise to the table
  - Helping stakeholders understand the impact of policies on undocumented students
  - Bringing data to the table as to the population of undocumented students past and present, and potential
  - Serving as outside agitators who can approach stakeholders without being involved in administrative reporting lines.

During the discussion, pose questions about bias in admissions, both conscious and unconscious. What processes, policies or practices are available to mitigate bias in admissions? Whose responsibility is it to ensure that the process is equitable? What can individuals engaging in admissions do if they identify a bias operating within the process or within a stakeholder? Encourage students to engage in scholarly practice by searching this topic to identify articles that provide information on best practices.

**Slide 21 – Case Outcome:**

Emphasize that student engagement and activism led to an article in Academic Medicine that provided guidance to residency programs and all medical schools regarding considering undocumented students. Three authors, all women of color: (Marquez Rojas was an M2, Rodriguez and Di Bartolo were both residents, Nakae was junior faculty)

Nakae S, Marquez DR, Bartolo IMD, Rodriguez R. Considerations for Residency Programs Regarding Accepting Undocumented Students Who Are DACA Recipients. *Academic Medicine*. 2017;92(11):1549-1554. doi:10.1097/acm.0000000000001731.

**Slide 22 – Case 2:**

- What faculty competencies apply to Dante?
  - Leading change
  - Communication skills
  - Leadership
- How can he advocate for continuous quality improvement or adaptation of the process?
  - Use his own experience
  - Cite specific examples
  - Present data that illustrates his point
  - Meet with stakeholders to prepare or rehearse his committee presentation in advance. Get buy in before finalizing the ‘ask.’

**Slide 23 – Case 2 Outcome:**

Describe the outcome of the case. Emphasize, again, the importance of leadership in effectuating an admissions process. Revisit any previous discussion points about mitigating bias and ways that individuals involved with the admissions process can contribute to ensuring equity.

**Slide 24 – Summary and Wrap Up:**

Summarize leadership competencies and opportunities.

**Slide 25 – Questions:**

If time allows, invite participants to ask questions.
