# Supplementary material for: Office of Admissions: Engagement and Leadership Opportunities for Trainees
Source: MedEdPORTAL. 2020 Nov 24;16:11018. doi: 10.15766/mep_2374-8265.11018 (PMC7703483; doi:10.15766/mep_2374-8265.11018)
Supplement: Supplementary file 1 — PowerPoint Presentation.pptxFacilitator Guide.docxPrereading Assignment.docxSkill-Set Group Mixer.docxAdmission Cases.docxPre- and Postworkshop Survey.docx [file mep_2374-8265.11018-s001.zip › C. Prereading Assignment.docx]

**Job Description for Assistant Dean for Admissions & Recruitment**

**Job Summary**

The Assistant Dean for Admissions and Recruitment (ADAR) will develop, implement, support, and evaluate the policies and procedures governing the comprehensive recruitment and admissions process of qualified students that reflect our Jesuit Catholic tradition and mission. The ADAR will assist the Dean with selecting, training, and managing all Admissions Committees, most especially the Executive Admissions Committee. The ADAR will report directly to the Associate Dean for Student Affairs while also responding to time sensitive requests directly from the dean. The ADAR will be committed to attracting diverse students with strong backgrounds in academic, service work, and a variety of life experiences. She/he will engage in systematic review and evaluation of all components of the admissions and matriculation process. The ADAR will supervise and coordinate the admissions unit staff and orchestrate/oversee recruitment and outreach initiatives. He/she will manage and update all aspects of recruitment including strategy, analysis of effectiveness, budget allocation, mission directives, media components online and in print, and relationship/affiliation building. The ADAR will serve as the chief contact with information technology to oversee the technical infrastructure of the admissions process and ensure all policies, procedures and safeguards are in place. The ADAR will serve as the lead contact for affiliated services such as MSAR and AMCAS to ensure the school remains current and compliant.

**Duties and Responsibilities**

- Supervises admissions staff to manage the admissions committees. This includes appointments, training, feedback, data metrics, communications, scheduling, and related work flow. The ADAR performs all necessary duties including: running selection committee meetings, interfacing and communicating with key faculty throughout each phase of the admissions process, interfacing with applicants, and all other duties related to the flow of the admissions process.
  - Serves as a key player in selecting emerging technologies to best support admissions and recruitment strategies. Communicates all changes and updates to IT infrastructure to communications team and ensures proper implementation/functionality.
  - Participates in developing, implementing, and supporting annual recruitment plan throughout the U.S., which includes traveling 1-2 times a month during the recruitment season.
  - Evaluates recruitment strategies for ROI using applicant and matriculant data utilizing data-driven metrics. May contribute to scholarly work in medical admissions community with a specific focus on mission-driven admissions, holistic review, and access/inequality in medicine.
  - Develops and fosters relationships with pre-health advisors, Program Directors, and faculty for the purpose of recruiting highly qualified and diverse applicants. Provides training and guidance to pre-health advisors through in person meetings, training sessions, and phone calls.
- Provides leadership for admissions within the institution and may participate in national and regional committees within the AAMC.
- Manages [or supervises director(s) who manage] the Executive Admissions Committee (EAC) and its proceedings in selecting applicants. Participates in EAC as ex-officio member. Ensures policies and procedures for the EAC are followed and updated as appropriate.
- Establishes and executes criteria for decisions that fall outside existing policies in concert with the Chair of the Executive Admissions Committee. Consults with faculty committees as needed.
- Supports and evaluates admissions mission, policies, and procedures. Works closely with faculty members to ensure all aspects of the process are consistent.
- Ensures all LCME accreditation standards are met regarding the admissions process, financial aid, matriculation process, and the confidentiality of student records.
- Manages and supports the Admissions database. Leads admissions staff in providing training to faculty and committee members who are database users.
- Manages and maintains the marketing materials for the Admissions department including the Admissions website, brochures, allied program information, etc.
- Manages information sent to and published by the AAMC including MSAR and other school specific policies/information.
- Demonstrates expertise in all phases of the admissions process including review of primary and secondary medical school applications, interviewing, selection, and matriculation.
- Manages the scholarship information and leads scholarship committee’s efforts in determining which accepted applicants are ideal for merit based scholarships.
- Creates and implements training for new Committee on Admissions members. Provides follow up support for committees and instructs new members on how to use evaluative metrics and technology for the process.
- Manages the department operating budget in close communication with the Associate Dean for Student Affairs.
- Supervises and mentors admissions staff members.
- Conducts annual performance reviews for admissions staff.
- Collaborates with colleagues and plans the annual Second Visit program for all accepted applicants.
- Ensures strong follow through and assistance for inquiries and applications regarding Deferred Action for Childhood Arrivals.
- Counsels and advises prospective students on the admissions process.
- Counsels and advises denied applicants.
- Offers support to Student Life team to review, evaluate, interview, and select participants and/or Teaching Assistants
- Develops and delivers presentations on critical aspects of admissions for pipeline student programs.
- Participates and engages in in student life activities to maintain an informed base for recruitment. Provides support to student groups and related events as relevant. (Latino Medical Student Association, Student National Medical Association, LGBTQA Association, and Asian Pacific American Medical Student Association, etc.)
- Manages application and selection process for transfer candidates with advanced standing and ensures smooth transfer to student affairs for admitted students.
- Manages application and selection process for dual degree students and ensures solid hand-off to appropriate units

**Reasoning and Ability**

Ability to define problems, collect data, establish facts, and draw valid conclusions. Ability to interpret an extensive variety of technical instructions in mathematical or diagram form and deal with several abstract and concrete variables.  Strong, proficient professional communication skills – both written and verbal. Ability to multitask and handle fast paced work environment. Ability to make decisions under pressure. Ability to apply policies fairly and equitably.

**Qualifications**

- Minimum qualification is Master's Degree with 5-7 years of admissions-related experience, MD, PhD, EdD or other terminal degree preferred.
- Academic medical center work, medical education experience, and enrollment management experience are highly desirable.
- Three to five years of managerial experience is preferred.
- Must possess excellent communication and presentation skills.
- Ability to articulate the mission of the University, and the ability to manage multiple tasks in a professional setting.
- A strong work ethic and desire to work with students from diverse backgrounds necessary.  
- Cultural humility and willingness to adapt and learn are critical.
- An understanding of social determinants of health, health disparities, community and underserved medicine are required.
