# Supplementary material for: Office of Admissions: Engagement and Leadership Opportunities for Trainees
Source: MedEdPORTAL. 2020 Nov 24;16:11018. doi: 10.15766/mep_2374-8265.11018 (PMC7703483; doi:10.15766/mep_2374-8265.11018)
Supplement: Supplementary file 1 — PowerPoint Presentation.pptxFacilitator Guide.docxPrereading Assignment.docxSkill-Set Group Mixer.docxAdmission Cases.docxPre- and Postworkshop Survey.docx [file mep_2374-8265.11018-s001.zip › D. Skill-Set Group Mixer.docx]

BNGAP: Office of Admissions Leadership and Engagement

Leadership Experiences Skill Set Mixer

| Served as officer in an organization | Served as RA in college | Participated in leading an orientation program | Worked as a tutor | Worked as a teaching assistant |
| --- | --- | --- | --- | --- |
| Established goals for a group | Established criteria for a program or award | Participated in group process for selecting or hiring | Supervised employees | Evaluated learners according to established criteria |
| Served as a peer counselor | Interviewed someone | Designed a process with workflows | Managed a budget | Facilitated a training |
| Evaluated an educational outcome | Evaluated selection criteria | Graded or provided feedback for assignments | Supervised volunteers | Gave a presentation |
| Provided advice | Provided emotional support | Mediated conflict | Challenged bias | Worked to change a policy |

Skill Set Mixer Leadership Competencies Key

**Self-management (SM):** demonstrates emotional intelligence in encounters with interviewees in a judicious and empathetic manner

**Working with and developing others (W):**  coaches peers in serving as effective tour guides, interviewers and committee members

**Leading change (LC):** understands change management through application of new or revised policies and procedures

**Communication skills (CS)**: creates a welcoming space to effectively convey information and listen to applicants

**Teambuilding (T)**: works with fellow admissions staff to problem solve and make decisions about applicants

**Leadership (L):** understands the structure and culture of the Office of Admissions

**Business skills (B):** evaluates and improves admissions policies and procedures through quality improvement efforts

| Served as officer in an organization (W, LC, CS, T, L, B) | Served as RA in college (SM, W, CS, L) | Participated in leading an orientation program (W, LC, CS, T, L, B) | Worked as a tutor (SM, W, CS) | Worked as a teaching assistant (SM, W, CS) |
| --- | --- | --- | --- | --- |
| Established goals for a group (SM, W, LC, CS, T, L) | Established criteria for a program or award (W, LC) | Participated in group process for selecting or hiring (W, CS, T, B) | Supervised employees (SM, W, CS, T, L, B) | Evaluated learners according to established criteria (SM, CS, L) |
| Served as a peer counselor (SM, CS, T) | Interviewed someone (SM, CS) | Designed a process with workflows (LC, L, B) | Managed a budget (L, B) | Facilitated a training (W, CS, L) |
| Evaluated an educational outcome (CS, L) | Evaluated selection criteria (CS, L) | Graded or provided feedback for assignments (SM, CS, L) | Supervised volunteers (SM, W, CS, T, L) | Gave a presentation (CS, L) |
| Provided advice (SM, CS, L) | Provided emotional support (SM, CS, L) | Mediated conflict (SM, W, CS, L) | Challenged bias (SM, W, CS, L) | Worked to change a policy (W, LC, CS, T, L) |

Lucas R, Goldman EF., Scott AR et al. Leadership Development Programs at Academic Health Centers: Results of a National Survey. Academic Medicine. 93(2):229-236, February 2018.)
