# Supplementary material for: Office of Admissions: Engagement and Leadership Opportunities for Trainees
Source: MedEdPORTAL. 2020 Nov 24;16:11018. doi: 10.15766/mep_2374-8265.11018 (PMC7703483; doi:10.15766/mep_2374-8265.11018)
Supplement: Supplementary file 1 — PowerPoint Presentation.pptxFacilitator Guide.docxPrereading Assignment.docxSkill-Set Group Mixer.docxAdmission Cases.docxPre- and Postworkshop Survey.docx [file mep_2374-8265.11018-s001.zip › E. Admission Cases.docx]

**Office of Admissions Engagement and Leadership Opportunities**

CASE 1:

You are an undocumented student who was able to navigate the uncertainty and difficulty of gaining admission to an MD program.

- - Which faculty competencies are crucial to be an effective advocate for future students? Please provide an example.
  - With whom would you strive to collaborate?
  - How does this align with LCME standards?
  - What skills are needed to adapt and respond to stakeholders?
  - What is the unique role students can play?

CASE 2:

Dante is a non-voting member of the executive admissions committee. He has served for two years and has been an active participant in discussions about candidate. Dante notices that the scoring system schemas make it so that one aberrant score from a single committee member can keep an applicant from getting an offer when the committee otherwise determines that they are an excellent mission fit.

- - What faculty competencies apply to Dante? Please provide examples.
  - How can he advocate for continuous quality improvement or adaptation of the process?
