# Supplementary material for: Office of Admissions: Engagement and Leadership Opportunities for Trainees
Source: MedEdPORTAL. 2020 Nov 24;16:11018. doi: 10.15766/mep_2374-8265.11018 (PMC7703483; doi:10.15766/mep_2374-8265.11018)
Supplement: Supplementary file 1 — PowerPoint Presentation.pptxFacilitator Guide.docxPrereading Assignment.docxSkill-Set Group Mixer.docxAdmission Cases.docxPre- and Postworkshop Survey.docx [file mep_2374-8265.11018-s001.zip › F. Pre- and Postworkshop Survey.docx]

**Pre-Evaluation Survey**

| **1. How much CONFIDENCE do you have in your ability to…** | **No Confidence**  **0** | **1** | **2** | **3** | **Complete**  **Confidence**  **4** |
| --- | --- | --- | --- | --- | --- |
| 1. Advocate for or facilitate change in the admissions process | 0 | 1 | 2 | 3 | 4 |
| 1. Engage in discussion about admissions policies and practices | 0 | 1 | 2 | 3 | 4 |
| **DEMOGRAPHICS:** | | | | | |
| 2. In which **STATE** is your medical school or residency program located? _____________________ | | | | | |
| 3. Are you a (circle one):   1. Medical Student 2. Intern/Resident 3. Fellow 4. Other (please specify):_______________________________ | | | | | |
| 4. What is your race/ethnicity (**circle all that apply)?**   1. American Indian or Alaska Native 2. Native Hawaiian or Other Pacific Islander 3. Asian 4. Black or African-American 5. Hispanic or Latino 6. White 7. Other (please specify): | | | | | |
| 5. How do you self-identify (**circle one**)?   1. Straight or Heterosexual 2. Gay or Lesbian 3. Bisexual 4. Other (please specify): ____________________ | | | | | |
| 6. How do you **self-identify**? Note: Respondents who self-identify as “Transgender female-to-male,” “Transgender male-to-female,” or “Transgender do not identify as exclusively male or female” are combined and displayed as “Transgender” (circle all that apply):   1. Male 2. Female 3. Transgender 4. Other: ________________________________ | | | | | |

**Post-Evaluation Survey**

| **1. How much CONFIDENCE do you have in your ability to…** | **No Confidence**  **0** | **1** | **2** | **3** | **Complete**  **Confidence**  **4** |
| --- | --- | --- | --- | --- | --- |
| 1. Advocate for or facilitate change in the admissions process | 0 | 1 | 2 | 3 | 4 |
| 1. Engage in discussion about admissions policies and practices | 0 | 1 | 2 | 3 | 4 |
| **2. To what extent do you agree that the workshop learning objectives were met** | **Strongly agree** | **Agree** | **Neither agree or disagree** | **Disagree** | **Strongly disagree** |
| 1. Describe the functions of the Office of Admissions | SA | A | N | D | SD |
| 1. Describe leadership competencies that are associated with various roles within the Office of Admissions | SA | A | N | D | SD |
| 1. Identify opportunities for trainees to engage in the Office of Admissions | SA | A | N | D | SD |
| 1. Review examples of how trainees can facilitate change | SA | A | N | D | SD |
| What did you like about this workshop? | | | | | |
| What suggestions do you have to improve this workshop? | | | | | |
