# Supplementary material for: Office of Admissions: Engagement and Leadership Opportunities for Trainees
Source: MedEdPORTAL. 2020 Nov 24;16:11018. doi: 10.15766/mep_2374-8265.11018 (PMC7703483; doi:10.15766/mep_2374-8265.11018)
Supplement: Supplementary file 1 — PowerPoint Presentation.pptxFacilitator Guide.docxPrereading Assignment.docxSkill-Set Group Mixer.docxAdmission Cases.docxPre- and Postworkshop Survey.docx [file mep_2374-8265.11018-s001.zip › _Educational_Summary_Report_11018.pdf]

MedEdPORTAL® The AAMC Journal of Teaching and Learning Resources

Original Publication Open Access

Office of Admissions: Engagement and Leadership Opportunities for Trainees

Sunny Nakae, PhD, MSW, Pooja Kothari, MD*, Karen Johnson, MD, Edgar Figueroa, MD, MPH, John Paul Sánchez, MD, MPH

*Corresponding author: poojapkothari12@gmail.com

Abstract

Introduction: Encouraging trainee engagement with the Office of Admissions can be an effective method of training for a future career in academic medicine and allow trainees to develop critical leadership skills. **Methods:** This workshop consisted of a short didactic presentation, a large-group activity, and case discussions in an effort to address four objectives describing the functions of the Office of Admissions, as well as identifying opportunities for involvement and leadership skills fostered through engaging in admissions activities. The module was administered to diverse students and residents at three regional conferences at US medical schools between September and December 2019. Pre- and postworkshop surveys were used to analyze the efficacy of the workshop. **Results:** More than 95% of the 70 learners agreed that all four objectives had been met. Additionally, trainees had a statistically significant increase ($p < .001$) in confidence in their ability to address new issues, such as Deferred Action for Childhood Arrivals or LGBT inclusion, through the admissions process and engage in discussion about admissions policies and practices. **Discussion:** This workshop was an effective tool for introducing trainees to leadership opportunities in academic medicine via involvement with the Office of Admissions. During the workshop, students expressed feedback about wanting more ways to become involved and more examples of student involvement. Attendees might also benefit from being encouraged to research the admissions processes and leadership structures at their respective institutions.

Keywords
Office of Admissions, Leadership, Academic Medicine Career Development, Admissions/Selection, Diversity, Inclusion, Health Equity, Leadership Development/Skills, Student Affairs

Educational Objectives

By the end of this activity, learners will be able to:

1. Describe the functions of the Office of Admissions.
2. Describe the skills and personal characteristics for admissions leadership roles.
3. Identify the opportunities for medical students to engage in the Office of Admissions.
4. Describe faculty leadership competencies facilitated through engagement in the Office of Admissions.

Introduction

Career development that encourages academic medicine careers is critical for the long-term success of academic medicine.

Citation:
Nakae S, Kothari P, Johnson K, Figueroa E, Sánchez JP. Office of Admissions: engagement and leadership opportunities for trainees. *MedEdPORTAL*. 2020;16:11018.
https://doi.org/10.15766/mep_2374-8265.11018

Medical student participation in activities with the Office of Admissions can be an accessible and effective training ground for careers in academic medicine. Unlike curriculum committees, where there may only be a few positions allocated for students, admissions tasks tend to present many opportunities for engagement due to the immense, constant, multiphased work of recruiting a class. Experiences with admissions activities from outreach and recruitment to the final selection committee can provide meaningful engagement and opportunities for initial or continued skill building that can lead to effective leadership and career development for academic medicine.

MedEdPORTAL features several publications to support the academic medicine career development of medical students, residents, and fellows, also referred to as prefaculty. Prior publications in *MedEdPORTAL* have focused on increasing trainees' understanding of the transparency of the appointment and promotion processes[1]; how to transform research, educational, and service-related work to scholarly products[2]; the value of mentorship in succeeding in academia[3]; and developing as a leader for a successful career in academia.[4] The

Copyright © 2020 Nakae et al. This is an open-access publication distributed under the terms of the Creative Commons Attribution license.

aforementioned modules largely focused on helping trainees gain a broad appreciation of faculty rank and responsibilities in relation to an academic department (e.g., clinical or basic science). The modules vaguely addressed faculty engagement with and responsibilities to medical school offices, another important layer to serving as a faculty member at a medical school. Our resource emphasizes how prefaculty, in their current roles as medical students, residents, and/or fellows, can become engaged and serve as leaders within medical school offices, laying a foundation of leadership competencies to eventually fulfill and succeed as a faculty member or senior administrator. To our knowledge, there are no other *MedEdPORTAL* publications focusing on admissions work and prefaculty career development.

This module was designed to help students understand opportunities for engagement in the Office of Admissions and the associated leadership skills required for careers in academic medicine. The module facilitates student exploration of leadership skills and faculty competencies related to engagement with the Office of Admissions. The module also serves to provide instruction about admissions leadership roles and the skills required, thereby facilitating medical student engagement in present activities and future leadership aspirations.

Building the Next Generation of Academic Physicians (BNGAP) is dedicated to developing workshops to heighten medical students' and residents' awareness of academic careers, especially for trainees underrepresented in medicine.[5] Dr. Sunny Nakae, a member of BNGAP's curriculum committee, created this workshop, which has been subsequently reviewed by a diverse team of students and faculty and continually refined to meet its stated goals and objectives. The current version of the workshop focuses on engagement with the Office of Admissions and the associated leadership skills that are foundational to faculty competencies. The five coauthors of the workshop have experience in admissions-related roles at their respective institutions. Their collective expertise is reflected in the workshop content and design.

BNGAP workshops also utilize the social cognitive career theory (SCCT) framework.[6] SCCT theorizes that in order to foster confidence in a career pursuit, learners must perceive high self-efficacy. Self-efficacy beliefs are generated based on exposure, role modeling, mentoring, and observation—the four tenets of Bandura's theory of self-efficacy.[7] The modules specifically attempt to provide exposure to the subject matter using faculty experts who can serve as role models and potential future mentors. Encouraging students' engagement in Office of Admissions activities will, in theory, bring opportunities for observation and reflection so that students can develop confidence in pursuing leadership roles in academic medicine in the future. This workshop has been designed to strengthen trainee self-efficacy by helping participants link past leadership experiences with faculty competencies. Participants also engage with real cases where medical student involvement in admissions activities led to positive change, which reinforces the efficacy of students as leaders in their prefaculty careers.

This workshop is part of several BNGAP offerings within a larger curriculum and can also be utilized as a singular module. Adaptations to this workshop should carefully consider the exposure, role modeling, mentoring, and observation framework and include these elements. Leadership development through admissions activities can serve to illuminate leadership pathways to develop the next generation of leaders for academic medicine.

Methods

We applied the six-step Kern model as a guiding framework for the design, implementation, and evaluation of the workshop.[8] An outline of how this framework was utilized follows.

1. Problem identification and general needs assessment: This workshop relied on existing literature establishing the need for greater support of trainees in pursuing careers in academic medicine, particularly trainees from diverse racial, sexual, and socioeconomic backgrounds.[9-11] The workshop development team incorporated active learning strategies and opportunities for personalization and advising, which were preferred learning modalities for trainees interested in academic careers.[12]
2. Targeted needs assessment: The workshop development team reviewed the literature and sought input from students and faculty regarding admissions committee engagement at multiple schools.
3. Goals and objectives: The overall goal of the workshop was to increase readiness and confidence for engagement with Office of Admissions activities. The learning objectives were met through personal story, case discussion, and an innovative mixer activity.[13]
4. Educational strategies: The material was designed using active learning such as case discussion and small-group activities. Small groups promoted learner engagement and facilitated active participation, purposeful activity, and meaningful interaction.[14]
5. Implementation: The 90-minute workshop was presented at three different medical schools with faculty facilitators and student and resident trainees. These medical schools volunteered to host the BNGAP Engagement

Copyright © 2020 Nakae et al. This is an open-access publication distributed under the terms of the Creative Commons Attribution license.

and Leadership in Academic Medicine Conference, at which this module was offered as one of the workshops. Learners were students and residents from presumably nearby areas, although every regional workshop was open to all. The conferences also facilitated professional networks and inter- and intraprofessional learning among students, staff, and faculty.

6. Evaluation and feedback: The workshop design and content were evaluated by conference participants using a pre/post design.

This workshop was designed for a diverse group of trainees, particularly for medical students, but also applicable to residents, fellows, and other health care professionals. The ideal facilitator was a health care professional in a leadership position, particularly with experience in the Office of Admissions of a health care professional graduate school and in small-group facilitation.

The workshop had several components, including a didactic PowerPoint presentation that described the functions of the Office of Admissions, the leadership skills important for different roles within the office, and opportunities for trainees to get involved with admissions. Embedded within the didactic portion were several activities, including discussion of a prereading assignment exemplifying the skills and qualities necessary for leadership within the Office of Admissions, a skill-set group mixer activity encouraging trainees to reflect on skills for leadership within admissions that they might already have acquired, and small-group discussion of two cases portraying real-life scenarios that leaders in the Office of Admissions could experience. The facilitator led a debrief on the cases to discuss how an actual leader within admissions addressed these scenarios and to explain the core leadership competencies those leaders used to this end. The resources required for this workshop are listed below.

- Appendix A: PowerPoint Presentation: The PowerPoint presentation served as the guide for the workshop, including the short didactic portion with information on the functions and roles of the Office of Admissions, opportunities for trainees to get involved, and examples of how trainees can affect change.
- Appendix B: Facilitator Guide: This step-by-step guide provided a detailed outline of what to discuss during the didactic and activity portions of this workshop, ensuring consistent implementation at a variety of sites.
- Appendix C: Prereading Assignment: This was a short description of the responsibilities of an admissions dean and the associated characteristics often sought in a qualified candidate. It was provided to attendees ahead of the workshop in order to expose them to what job descriptions for leadership roles in admissions looked like. Participants were asked to examine their current credentials against the job description. What experiences might they need to seek out to become qualified for the position? Discussing this at the beginning of the workshop allowed trainees to become more familiar with the Office of Admissions and requirements for engagement.
- Appendix D: Skill-Set Group Mixer: This worksheet listed several important experiences and qualities typically seen on American Medical College Application Service applications of student applying to medical school that were also important for a member of the Office of Admissions. The goal of this activity was to demonstrate to trainees that many of the qualities and skills that they currently were developing or already possessed overlapped with those of professionals working within admissions.
- Appendix E: Admission Cases: This handout provided two real-life scenarios exemplifying challenges that could arise in the Office of Admissions and the qualities that helped a professional within the office address these challenges.
- Appendix F: Pre- and Postworkshop Survey: These surveys asked trainees questions that assessed their prior knowledge of the Office of Admissions and evaluated the efficacy of the workshop. The postworkshop portion also provided an opportunity for trainees to discuss what they liked about the training and suggestions on how to improve the experience.

One or two cofacilitators administered the workshop and allotted 2-3 hours to review the content of the training in advance. In order to properly conduct the workshop, pens, audiovisual equipment for the PowerPoint presentation, chairs and tables arranged to facilitate small-group discussion for three to seven participants, and printed copies of the worksheets and evaluation forms were provided. The suggested length of the workshop was 66 minutes, with an outline below:

- Preworkshop evaluation—3 minutes.
- Slides 1-13—20 minutes.
- Slides 14-16: group activity and discussion—15 minutes.
- Slides 17-19—10 minutes.
- Slides 20-25: case discussions—12 minutes.
- Slide 26: questions and answers—3 minutes.
- Postworkshop evaluation—3 minutes.

Copyright © 2020 Nakae et al. This is an open-access publication distributed under the terms of the Creative Commons Attribution license.

Results

This workshop was implemented at three conference sites: Weill Cornell Medical College, University of Oklahoma College of Medicine, and McGovern Medical School. A total of 88 trainees completed partial or full workshop evaluations. The workshop was facilitated by a total of three presenters, including an interim assistant vice provost for academic affairs, an associate professor of family medicine, and an associate dean of admissions.

Demographics of the 82 conference attendees who responded to the preconference survey are provided in Table 1.

Additionally, 77 attendees answered the question "How knowledgeable are you in identifying leadership opportunities for trainees to become engaged through the Office of Admissions?" In response, 39 attendees (51%) replied *not knowledgeable,* 27 (35%) replied *somewhat knowledgeable,* and 11 (14%) replied *knowledgeable.* To assess attendees' background experience, they were also asked to identify if they had "participated on a committee or task force overseen by the Office of Admissions or its equivalent" during several time periods. Sixteen percent of attendees responded that they had prior to medical school, 58% during medical school or prior to residency, 14% during residency, and 5% after residency. For the same categories, 8%, 8%, 87%, and 93%, respectively, noted that this question was not applicable to them due to their level of training.

Table 1. Demographics of 82 Respondents

Demographic	No. of Respondents (%)
Education level	
Medical student	65 (79)
Intern/resident	7 (8)
Fellow	3 (4)
Other	7 (8)
Race/ethnicity[a]	
Native American or Alaska Native	3 (4)
Native Hawaiian or Pacific Islander	1 (1)
Asian	15 (18)
Black or African American	26 (32)
Hispanic or Latino	19 (23)
White	25 (30)
Other	3 (4)
Gender	
Male	35 (43)
Female	47 (57)
Transgender	0 (0)
Other	0 (0)
Sexual orientation	
Straight or heterosexual	70 (86)
Gay or lesbian	5 (6)
Bisexual	6 (7)
Other	0 (0)

[a]Participants were encouraged to select more than one race/ethnicity they identified with.

A paired sample *t* test ($p < .05$) was used to compare pre- and postworkshop survey responses. Seventy of the 82 attendees responded to both pre- and postworkshop surveys. Participants reported a statistically significant increase in confidence regarding the following statements: "Address new issues, such as Deferred Action for Childhood Arrivals or LGBT inclusion, through the admissions process," and "Engage in discussion about admissions policies and practices." Results are exhibited in Table 2.

Additionally, efficacy of the workshop was determined by asking attendees, "To what extent do you agree that the workshop learning objectives were met?" For all four objectives, over 95% of attendees either agreed or strongly agreed that the objectives had been met.

Comments on the workshop were collected to identify positive areas of the curriculum, as well as suggestions on how to further improve the module. Sample comments below are organized by learning objectives.

Responses to the question "What did you like about this workshop?" included the following:

- Objective 1: Describe the functions of the Office of Admissions.
  - "I liked that the speaker talked about all the roles of deans for Office of Admissions and all the training needed to be a dean."
- Objective 2: Describe the skills and personal characteristics for admissions leadership roles.
  - "Table of experiences that is 'prep work' of skills needed to be a successful academic physician in admissions. I greatly appreciated the encouragement that the speaker gave for feats, large and small."
  - "Engaging discussion and facilitation. Examples of qualities needed for leading in admissions."
- Objective 3: Identify the opportunities for medical students to engage in the Office of Admissions.

Table 2. Results for Pre- and Postworkshop Survey Responses

Question	Average[a]		p[b]
	Pre	Post	
How much confidence do you have in your ability to:			
Address new issues, such as Deferred Action for Childhood Arrivals or LGBT inclusion, through the admissions process	2.0	2.9	<.001
Engage in discussion about admissions policies and practices	1.8	3.2	<.001

[a]A 4-point Likert scale (0 = *no confidence,* 4 = *complete confidence*) was used to assess responses.
[b]A paired sample *t* test was used to assess statistically significant change ($p < .05$).

Copyright © 2020 Nakae et al. This is an open-access publication distributed under the terms of the Creative Commons Attribution license.

  - "Informative, handout was great, great description of opportunities in Office of Admissions."
  - "Described responsibilities and opportunities within admissions."
- Objective 4: Describe faculty leadership competencies facilitated through engagement in the Office of Admissions.
  - "I liked how much it was iterated that students can be involved, and future opportunities in admission."
  - "This workshop was an eye opener to how we can make a difference in admissions if we become involved."

Responses to the question "What suggestions do you have to improve this workshop?" included the following:

- Objective 1: Describe the functions of the Office of Admissions.
  - "Provide more examples on how to be involved in admission committee and the roles found in the Office of Admissions. Not many examples were explored. Also, not great detail was put into explaining the role of Office of Admissions."
  - "I think there could be more description (i.e., flow charts) to describe the basic structure of an Office of Admissions."
- Objective 2: Describe the skills and personal characteristics for admissions leadership roles.
  - "I wanted to hear more about the qualities that admissions offices look for in particular."
- Objective 3: Identify the opportunities for medical students to engage in the Office of Admissions.
  - "More details on how students can impact admissions would help me get more involved."
  - "More examples of student opportunities to get involved."
- Objective 4: Describe faculty leadership competencies facilitated through engagement in the Office of Admissions.
  - "More specific examples for residents on how to get involved."

Discussion

To encourage trainee involvement in academic medicine, we developed this workshop specifically focused on one area where medical students may have opportunities for engagement: admissions. Participants were introduced to leadership competencies for future faculty roles and basic tenets of admissions in allopathic medicine. After active exploration of their previous engagement in leadership, participants discussed cases and learned of instances where medical students engaged in scholarship as a result of engagement.

Attendees provided excellent feedback that aided the workshop team in refining the workshop's design and elements. Across all four learning objectives, 95% of attendees across all three sites agreed or strongly agreed that the workshop objectives had been met. To improve the first learning objective, we emphasized that facilitators should showcase their local admissions process and provide specific organizational charts as examples on slides 14 and 18 of the PowerPoint (Appendix A). This emphasis was added to the facilitator guide (Appendix B).

For the second objective, we included a description of seven leadership competencies of leaders in the Office of Admissions that provided a tangible takeaway to reinforce material presented in the PowerPoint slides. These competencies, combined with the skill-set group mixer (Appendix D), emphasized leadership skills while helping students identify activities in which they might already have engaged. This was meant to increase confidence in skills for future engagement, which was consistent with the SCCT framework that served as the scaffold for this workshop.

During the workshop, students expressed feedback about wanting more ways to become involved and more examples of student involvement (the third and fourth objectives). We encourage facilitators to emphasize these aspects whenever possible. The facilitator guide includes notes about the benefit of using local examples and providing specific information about admissions committee involvement. Attendees might also benefit from being encouraged to research the admissions processes and leadership structures at their particular institutions. Facilitators could encourage attendees to reach out to leadership and inquire about involvement with the Office of Admissions as a follow-up to the session.

Limitations

The limitations of the workshop can be summarized in four areas: sampling, consistency, outcomes, and generalizability. Our sample of attendees was drawn across three sites and included only a small number of residents and fellows. Satisfaction with learning objectives and workshop deliverables was largely based on medical student input, as that was the majority of the sample. While we believe this workshop is effective with graduate medical education learners, there was limited sampling of this group in our attendee population. The workshops were conducted at BNGAP academic medicine conferences, and therefore, the samples constituted individuals predisposed to interest in academic careers. This may have influenced the satisfaction ratings since these attendees were likely enthusiastic about academic medicine.

Copyright © 2020 Nakae et al. This is an open-access publication distributed under the terms of the Creative Commons Attribution license.

Consistency in content delivery was another limitation of this workshop. The BNGAP curriculum team made every effort to create modules balancing personalization of the facilitator's experience (a key element of social learning theory) and standardization of learning objectives (an important aspect of the BNGAP series). Our sample was collected across three sites, and our data were analyzed in the aggregate. We were not able to differentiate if one site was better than another at meeting the learning objectives or had higher learner satisfaction. We did not collect data on individual facilitators or assess facilitator consistency since each site was an independent observation.

This workshop was primarily designed to promote diverse prefaculty awareness of engagement with and leadership opportunities in the Office of Admissions, as well as of leadership competencies developed along the way. Given the brief nature of this career-related educational module, we limited the evaluation to the level 1 reaction of Kirkpatrick's evaluation model.[15] As such, this module is a short-term intervention and not conducive to assessing long-term outcomes. We are confident that the information provided advances the career goals and interests of trainees. We are able to report their levels of satisfaction and whether or not they believed the module's objectives had been met. We were not able to make another observation of the outcomes of participants related to their participation in the Office of Admissions following engagement with this curriculum. Incorporation of this module into a longitudinal program can allow for additional evaluation at the levels of learning, behavior, and results.

Finally, we recognize that medical school admissions processes and structures differ across allopathic medical schools. Trainees may not have opportunities to engage with or contribute to the admissions process at their respective institutions. This curriculum can still serve to raise awareness about admissions roles and illustrate ways that trainees can be involved. Perhaps this curriculum could inspire trainees to approach leadership in order to facilitate involvement at their institutions if involvement is not yet a norm. Furthermore, trainees who become aware of the possibilities and scope for trainee involvement in admissions may seek leadership opportunities in academic medicine for their careers. As future leaders, they may be able to incorporate trainees in admissions processes in the future.

This workshop is an effective tool for introducing trainees to leadership opportunities in academic medicine via involvement with the Office of Admissions. Evaluative metrics demonstrate that the design and content of this workshop successfully fulfilled the learning objectives. The BNGAP leadership team aims to produce content upon which facilitators can rely to develop strong leaders in academic medicine. The content has been thoughtfully designed to appeal to trainees from all backgrounds. By specifically connecting medical students and residents with opportunities for engagement in the Office of Admissions and linking their skills to faculty competencies, trainees will gain both knowledge and confidence to pursue academic careers.

Appendices

A. PowerPoint Presentation.pptx
B. Facilitator Guide.docx
C. Prereading Assignment.docx
D. Skill-Set Group Mixer.docx
E. Admission Cases.docx
F. Pre- and Postworkshop Survey.docx

All appendices are peer reviewed as integral parts of the Original Publication.

Sunny Nakae, PhD, MSW: Associate Dean for Student Affairs and Clinical Associate Professor of Social Medicine, University of California, Riverside, School of Medicine

Pooja Kothari, MD: Internal Medicine Resident, Montefiore Medical Center

Karen Johnson, MD: Associate Dean for Admissions and Associate Professor of Pediatrics, Baylor College of Medicine

Edgar Figueroa, MD, MPH: Associate Professor of Family Medicine in Clinical Medicine, Weill Cornell Medical College

John Paul Sánchez, MD, MPH: President, Building the Next Generation of Academic Physicians; Executive Director, Latino Medical Student Association

Acknowledgments
We would like to acknowledge Brandt Wiskur, MSW, PhD, Interim Assistant Vice Provost for Academic Affairs, University of Oklahoma Health Sciences Center.

Disclosures
None to report.

Funding/Support
None to report.

Ethical Approval
The Rutgers University Health Sciences Institutional Review Board approved this study.

Copyright © 2020 Nakae et al. This is an open-access publication distributed under the terms of the Creative Commons Attribution license.

References

1. Callahan EJ, Banks M, Medina J, Disbrow K, Soto-Greene M, Sánchez JP. Providing diverse trainees an early and transparent introduction to academic appointment and promotion processes. *MedEdPORTAL*. 2017;13:10661. https://doi.org/10.15766/mep_2374-8265.10661
2. Paredes Molina CS, Spencer DJ, Morcuende M, et al. An introduction to research work, scholarship, and paving a way to a career in academic medicine. *MedEdPORTAL*. 2018;14:10686. https://doi.org/10.15766/mep_2374-8265.10686
3. Ortega G, Smith C, Pichardo MS, Ramirez A, Soto-Greene M, Sánchez JP. Preparing for an academic career: the significance of mentoring. *MedEdPORTAL*. 2018;14:10690. https://doi.org/10.15766/mep_2374-8265.10690
4. Dickerman J, Sánchez JP, Portela-Martinez M, Roldan E. Leadership and academic medicine: preparing medical students and residents to be effective leaders for the 21st century. *MedEdPORTAL*. 2018;14:10677. https://doi.org/10.15766/mep_2374-8265.10677
5. Building the Next Generation of Academic Physicians. 2020. http://bngap.org/
6. Lent RW, Brown SD, Hackett G. Contextual supports and barriers to career choice: a social cognitive analysis. *J Couns Psychol*. 2000;47(1):36-49. https://doi.org/10.1037/0022-0167.47.1.36
7. Bandura A. Self-efficacy. In: Weiner IB, Craighead WE, eds. *The Corsini Encyclopedia of Psychology*. Wiley; 2010:1534.
8. Thomas PA, Kern DE, Hughes MT, Chen BY, eds. *Curriculum Development for Medical Education: A Six-Step Approach*. 3rd ed. Johns Hopkins University Press; 2016.
9. Sánchez JP, Peters L, Lee-Rey E, et al. Racial and ethnic minority medical students' perceptions of and interest in careers in academic medicine. *Acad Med*. 2013;88(9):1299-1307. https://doi.org/10.1097/ACM.0b013e31829f87a7
10. Greenberg RB, Ziegler CH, Borges NJ, Elam CL, Stratton TD, Woods S. Medical student interest in academic medical careers: a multi-institutional study. *Perspect Med Educ*. 2013;2(5-6):298-316. https://doi.org/10.1007/s40037-013-0051-6
11. Sánchez NF, Rankin S, Callahan E, et al. LGBT trainee and health professional perspectives on academic careers—facilitators and challenges. *LGBT Health*. 2015;2(4):346-356. https://doi.org/10.1089/lgbt.2015.0024
12. Sánchez JP, Gemeda M, Guilliames CE, et al. Interventions to increase and maintain diverse medical student interest in academic careers. Presented at: GDI/GSA/OSR National Meeting; April 26-29, 2014; San Diego, CA.
13. Lucas R, Goldman EF, Scott AR, Dandar V. Leadership development programs at academic health centers: results of a national survey. *Acad Med*. 2018;93(2):229-236. https://doi.org/10.1097/acm.0000000000001813
14. van Blankenstein FM, Dolmans DHJM, van der Vleuten CPM, Schmidt HG. Which cognitive processes support learning during small-group discussion? The role of providing explanations and listening to others. *Instr Sci*. 2011;39(2):189-204. https://doi.org/10.1007/s11251-009-9124-7
15. Kirkpatrick's four-level training evaluation model: analyzing learning effectiveness. *MindTools*. Accessed October 14, 2020. https://www.mindtools.com/pages/article/kirkpatrick.htm

Received: January 26, 2020
Accepted: July 9, 2020
Published: November 24, 2020

Copyright © 2020 Nakae et al. This is an open-access publication distributed under the terms of the Creative Commons Attribution license.
